# Overexpression of the *Tectona grandis TgNAC01* regulates growth, leaf senescence and confer salt stress tolerance in transgenic tobacco plants

**DOI:** 10.7717/peerj.13039

**Published:** 2022-03-03

**Authors:** Fernando Matias, Perla Novais de Oliveira, Olman Gómez-Espinoza, Esteban Galeano, Helaine Carrer

**Affiliations:** 1 Department of Biological Sciences, Luiz de Queiroz College of Agriculture (ESALQ), Universidade de São Paulo, Piracicaba, São Paulo, Brasil; 2 Laboratory of Physiology and Plant Molecular Biology, Agroindustry Institute, Universidad de La Frontera, Temuco, Chile; 3 Department of Renewable Resources, University of Alberta, Edmonton, Alberta, Canada

**Keywords:** Tropical tree physiology, Plant stress, Functional analysis, Transcription factor

## Abstract

NAC transcription factors play critical roles in xylem secondary development and in regulation of stress response in plants. NAC proteins related to secondary cell wall development were recently identified and characterized in *Tectona grandis* (teak), one of the hardwood trees of highest economic importance in the world. In this work, we characterized the novel *TgNAC01* gene, which is involved in signaling pathways that mediate teak response to stress. Abscisic acid (ABA) increases *TgNAC01* expression in teak plants. Therefore, this gene may have a role in signaling events that mediate ABA-dependent osmotic stress responsive in this plant species. Stable expression in tobacco plants showed that the TgNAC01 protein is localized in the cell nucleus. Overexpression of *TgNAC01* in two out three independent transgenic tobacco lines resulted in increased growth, leaf senescence and salt tolerance compared to wild type (WT) plants. Moreover, the stress tolerance of transgenic plants was affected by levels of *TgNAC01* gene expression. Water potential, gas exchange and chlorophyll fluorescence were used to determine salt stress tolerance. The 35S:TgNAC01-6 line under 300 mM NaCl stress responded with a significant increase in photosynthesis rate, stomatal conductance, transpiration and carboxylation efficiency, but lower water potential compared to WT plants. The data indicate that the *TgNAC01* transcription factor acts as a transcriptional activator of the ABA-mediated regulation and induces leaf senescence.

## Introduction

The vascular system plays an essential role in adaptive mechanisms of land plants by providing mechanical support and transport of water, nutrients, hormones and other signaling molecules (De Rybel et al., 2016). The adaptive processes involve organization of tissues and cells that are regulated by signal molecules, such as hormones and transcription regulatory networks. Among the signal molecules, NAC and MYB proteins are crucial for the organization of vascular tissues (Nakano et al., 2015). NAC has been reported as one of the largest TF families in plants. Its designation is derived from the *N**O* *A**PICAL* *M**ERISTEM* (*NAM*) gene from *Petunia hybrida* and the *Arabidopsis thaliana ATAF1–2* and *CU**P-SHAPED* *C**OTYLEDON* 2 (*CUC2*) genes (Ooka et al., 2003). Members of this protein family have important functions in senescence regulation (Kim et al., 2018; Li et al., 2018), cell division, wood formation (Zhong, Lee & Ye, 2010a; Zhong, Lee & Ye, 2010b), and biotic and abiotic stress responses (Zhang et al., 2018).

Differentiation of xylem cells during wood formation undergo drastic cytological changes that have been extensively studied (Ruzicka et al., 2015; Zhong, Cui & Ye, 2019). Vessels and fibers form the secondary xylem through developmental processes that include cell division, cell expansion, secondary wall deposition, lignification and programmed cell death (Zhong et al., 2008; Zhong, Cui & Ye, 2019). These biological processes are controlled by a sophisticated network of transcription factors (TFs) that coordinately integrate developmental and environmental signals in the secondary cell wall (SCW) formation (Taylor-Teeples et al., 2015). A particular group of NAC transcription factors, denominated VNS proteins, regulates wood formation. VNS proteins refer to *V**ASCULAR RELATED* *N**AC-**D**OMAIN* 1-7 *(VND1-7)* (Zhong, Lee & Ye, 2010b), *N**AC* *S**ECONDARY WALL* *T**HICKENING PROMOTING FACTOR* 1 *AND* 3 *(NST1/3))* (Mitsuda et al., 2007), *S**ECONDARY WALL-ASSOCIATED* *N**AC* *D**OMAIN PROTEIN* 1-2 *(SND1-2)* (Zhong, Demura & Ye, 2006) and *SMB (Sombrero)* (Kamiya et al., 2016). In the *Arabidopsis* regulatory network for secondary cell wall biosynthesis, *ANAC075* is a putative regulator of *VND7* (Fujiwara & Mitsuda, 2016) and *V**ASCULAR* *N**AC* *I**NTERACTING* 2 (*AtVNI2*) is a negative regulator of *VND7* (Yamaguchi et al., 2010).

Modifications of *NAC* gene expression patterns have resulted in transcriptional changes and stress tolerance in several plant species (Nuruzzaman, Sharoni & Kikuchi, 2013). Under salt stress, *AtVNI2* expression has been related to ABA-mediated response of leaf senescence by regulating the genes *CO**LD-**R**EGULATED* (*COR*) and *R**ESPONSIVE TO* *D**EHYDRATION* (*RD*) (Yang et al., 2011). Other NAC proteins have been associated with stress response. For instance, expression of *ANAC032*, an abscisic-acid-responsive *NAC* gene of Arabidopsis, was *induced* under diverse stress conditions, such as oxidative, light, osmotic and salinity stress in comparison to control plants (Mahmood et al., 2016). Overexpression of *ANAC019*, *ANAC055* and *ANAC072* significantly enhanced drought tolerance in this plant species (Li et al., 2012). Besides, the *NAC57* gene of *Populus trichocarpa* overexpressed in Arabidopsis improved salt stress tolerance of transgenic plants (Yao et al., 2018). Furthermore, overexpression of the *Populus euphatica PeNAC036* gene in Arabidopsis increased plant tolerance to salt stress and drought (Lu et al., 2018).

The *NAC* TF family has been recognized to play a significant role in leaf senescence regulation (Podzimska-Sroka et al., 2015). About half of the known Arabidopsis NAC members (65 of 113 genes) undergo expression changes during leaf aging (Kim et al., 2018). In leaf senescence, the *ANAC016* (Podzimska-Sroka et al., 2015), *ANAC029/AtNAP*, *ANAC059/ORS1* and *ANAC092/ORE1* genes were characterized as positive NAC regulators, whereas the *ANAC042/JUB1* and *ANAC083/ AtVNI2* genes were negative regulators (Yang et al., 2011; Kim, Nam & Lim, 2016). Leaf aging is usually accompanied by transcriptional reprogramming of a large number of genes named *S**enescence-* *A**ssociated* *G**enes* (*SAGs*). Moreover, ABA-signaling activation of NAC TFs is associated with physiological responses during leaf aging. The ANAC046 TF a positive regulator of *Arabidopsis* leaf senescence by controlling the expression of chlorophyll catabolic genes and other senescence-associated genes (Oda-Yamamizo et al., 2016).

As for *Tectona grandis* (teak), recent transcriptome and genome studies have also included secondary xylem biosynthesis. A chromosomal-scale genome assembly of teak allowed characterization of the terpene synthase protein family, which is involved in the defense of woody tissues against pests and pathogens (Zhao et al., 2019). Previously, transcriptome proteins were assembled in lignified tissues of teak (Galeano et al., 2015). Subsequently, the TgNAC01 teak transcription factor was described as orthologous of the AtVNI2 protein with the highest relative expression in sapwood in comparison to other tissues (Camel, Galeano & Carrer, 2017). Also, seven AtVNI2 orthologous proteins of *Nicotiana tabacum* (Li et al., 2018), six paralogous of *TgNAC01* and one orthologous of *ANAC083* (*AtVNI2*) from Arabidopsis, which are related to secondary growth, were recently identified in teak (Hurtado et al., 2020).

The objective of the present work was to broaden the understanding of the *TgNAC01* transcription factor of *Tectona grandis*. Phylogenetic analysis of this gene and transgenic tobacco lines were generated to study subcellular localization and the role of TgNAC01 protein in leaf senescence and salt stress response. In addition, analyses of relative expressions of different elicitor-stress in teak plants grown *in vitro* and physiological analyses of transgenic tobacco indicated that *TgNAC01* acts as a transcriptional activator of the stress mediated regulation.

## Materials and Methods

### Phylogenetic analysis and evolutionary profile of the *TgNAC01* gene

The TgNAC01 protein sequence was obtained from teak transcriptome (NCBI accession number MH003855.1) (Galeano et al., 2015) and aligned to previously identified teak NAC proteins (Hurtado et al., 2020) using MAFFT v7.407 (Katoh & Standley, 2013) in auto mode. A phylogenetic tree with 116 teak NAC proteins and the TgNAC01 transcription factor was constructed using MEGA 7.0 (Kumar et al., 2018). In addition, an unrooted phylogenetic tree was set with 25 homologous proteins from 22 species, including Clorophyta, Bryophytas, Lycophytas, Gimnosperms and Angiosperms, and selected using BLASTp in the Plant Transcription Factor DataBase v.5.0 (Jin et al., 2017), following the highest scores and lowest *E*-value per species. The initial tree was obtained as a matrix of pairwise distances using Neighbor-Joining (NJ), BioNJ algorithms and the JTT model. The topology was selected with superior log likelihood value. The maximum-likelihood method and Poisson correction model with 1000 replicates were used to represent the evolutionary history of the taxa. Protein structure prediction in monomer and dimer forms was performed with AlphaFold2 (Jumper et al., 2021). The MEME v5.0.5 software (Bailey et al., 2009) was employed for the detection of conserved motifs using 20 maximum number of motifs and a range of ≥6 and ≤25 for optimum motif width. The FIMO v. 5.1.1 motif database (Bailey et al., 2009) was used for motif search in the auto mode option.

### Plant material and stress treatments

Teak seedlings were grown in glass flasks containing 30 mL half-strength MS (Murashige and Skoog; Sigma-Aldrich, St. Louis, MO, USA) (pH 5.8, 1% agar) medium and incubated at 25 °C under long-day conditions (16 h light/8 h dark) and 45 µmol photons m^−2^ s^−1^ PAR irradiance. As for salt stress treatments, 60-day-old teak seedlings were transplanted to a fresh MS media supplemented with 150 mM NaCl (high salinity), 20% PEG (high osmotic pressure), 100 µM methyl jasmonate (MeJA) (Bedoukian Research Inc., Danbury, CT, USA) or 100 µM ABA (Sigma-Aldrich). Leaf samples were collected at 0 h, 3 h, 6 h and 12 h of stress treatment and immediately frozen in liquid nitrogen for RNA extraction and qRT-PCR experiments.

### Construction of the *TgNAC01* overexpression vector and plant transformation

The full-length coding sequence (CDS) of *TgNAC01* was amplified using previously synthetized cDNA from teak secondary xylem and a set of primers containing the “CACC” sequence in the N-terminal region (Table S1). In a first construct, the stop codon from the amplicon was excluded to allow the expression of the *EGFP* reporter gene (Green Fluorescent Protein) in the pK7FWG2 destination vector in order to observe the *TgNAC01* subcellular localization. A second construct containing the stop codon was prepared and the *TgNAC01* gene was cloned into the pENTR^™^/D-TOPO^®^ cloning vector. Additionally, a plasmid recombination was performed *via* LR clonase in the pK7WG2 destination vector using the gateway system with the CaMV35S promoter following the manufacturer’s instructions (Thermo Fisher, Waltham, MA, USA). The position and integrity of DNA sequences in the vectors were confirmed by digestion with restriction enzymes, DNA electrophoresis, PCR and DNA sequencing. The two vectors containing p35S:TgNAC01 were used to transform the EAH105 strain of *Agrobacterium tumefaciens*. In parallel, *Nicotiana tabacum* seedlings (cv. Petit Havana SR1) were grown in glass jars with half concentration of MS salts, under 16/8 h light/dark photoperiod, at 25 °C, and used as control plants (WT) in the experiments. Leaf discs from 30-day-old WT tobacco plants were used as explants for *Agrobacterium*-mediated transformation. Tobacco leaf disks were immersed into a cell suspension (OD_600_ nm = 0.5–0.8) of the *A. tumefaciens* harboring the gene of interest, incubated for 20 min and then co-cultivated for 2 days in the dark. Cultures were cultivated under 16/8 h light/dark photoperiod at 25 °C and subcultured in a 3 week-interval.

### Subcellular localization of proteins

The *TgNAC01* gene lacking the stop codon was cloned in frame into the pK7FWG2 vector with the EGFP gene at the C-terminal portion and named 35S:TgNAC01-EGFP. The pK7FGW2 empty vector, named 35S:EGFP, was used as control. The vectors were used for genetic transformation of thirty-day-old tobacco leaves *via Agrobacterium tumefaciens* EAH105 strain (Horsch et al., 1984). Fluorescence visualization was checked in the root differentiation zone of 7-day-old transgenic tobacco lines (T1-first generation) using a FV1000 Olympus FluoView microscope (Olympus, Tokyo, Japan) with an argon laser. Excitation wavelength was calibrated between 488 and 509 nm. Protein subcellular localization prediction was determined with CELLO v.2.5 (http://cello.life.nctu.edu.tw/), WoLF PSORT (https://wolfpsort.hgc.jp/), and LocTree3 (https://rostlab.org/services/loctree3). In addition, HMMTOP (http://www.enzim.hu/hmmtop/index.php) was used for transmembrane prediction.

### Characterization of *TgNAC01* tobacco transgenic lines

The second generation (T2) of the tobacco *TgNAC01* transgenic lines was screened from sowed seeds on half strength MS medium supplemented with 100 mg L^−1^ Kanamycin (Kan). Green lines segregating at 3:1 ratio (Kan-resistant:Kan-susceptible) were recognized as transgenic lines. The 100% kanamycin resistant T2 seeds germinated on selective medium were selected as homozygous transgenic lines. Total DNA was isolated from selected lines using the CTAB method. Transgenic lines were confirmed by PCR using specific primers (Table S1) for amplification of the *TgNAC01* and *neomycin phosphotransferase II* (*nptII*) genes.

### Salt stress treatment in transgenic tobacco plants

Transgenic tobacco lines were grown in a soil and substrate mix contained in 5L pots, under maximum luminosity incidence of 200 µmol photons m^−2^ s^−1^ (at noon), 27 °C average temperature and about 60% humidity controlled by an automatic water curtain system in the greenhouse. Relative water content (RWC) in the pots was estimated using the gravimetric method as the difference between the soil weight after drainage and soil weight after drying. Irrigation water volume was calculated from the relative water content and evapotranspiration data, following the Penman-Monteith equation. The tobacco crop coefficient (Kc) corresponding to a value of 0.8 was calculated according to previous studies (Rao, 2012). Plants were watered with 0, 150 and 300 mM NaCl aqueous solution for seven days. Experiments consisted of three independent biological replicates for each treatment. The second fully expanded leaf from the apical meristem was collected from each plant for gene expression analysis.

### Leaf water potential and gas exchange measurements in transgenic tobacco plants

The average water potential was measured in one leaf of the fourth pair of fully expanded leaves at 4:00 a.m. (predawn) and 1:00 p.m. (midday) using a pressure chamber (PMS Instrument Company, Model 1000, Albany SE, USA). The relative water content was calculated as RWC = (fresh weight − dry weight)/(turgid weight − dry weight) ×100 (Pieczynski et al., 2013). Physiological parameters net CO_2_ assimilation (photosynthesis) rate (A), stomatal conductance to water vapor (gs), transpiration (E), and carboxylation efficiency (A/Ci) were measured at day 7 of salinity stress. All measures were done in the second pair of fully expanded leaves below the apex using three replicates. Instantaneous water use efficiency (WUEins, mmol mol^−1^) was defined as the A/E ratio and intrinsic water use efficiency (WUEint, µmol mol^−1^) as the A/gs ratio (Sinclair, Tanner & Bennett, 2013). All leaf gas exchange measurements were performed between 08:00 a.m. and 12:00 p.m., at 25 °C, with a portable infrared CO_2_/H_2_O gas analyzer (IRGA) (LCpro-32 070, ADC Bioscientific Ltd., Great Amwell, U.K.) equipped with a broad leaf chamber. The Photosynthetic Response to Light Intensity (P.R.L.I) was measured on leaves exposed to 400 ppm CO_2_ using Photosynthetically Active Radiation (P.A.R.) of 1,200 µmol m^−2^ s^−1^ at 25 °C.

### Chlorophyll and fluorescence measurements

Chlorophyll fluorescence was measured from 8:30 a.m. to 12:00 p.m. using a portable JUNIOR-PAM Chlorophyll fluorometer (Walz, Effeltrich, Germany). Quantum efficiency of PSII (ΦPSII) was assessed with a Saturation Pulse (10,000 µmol quanta m^−2^ s^−1^, 800 ms) at 3 min illumination with 1,050 µmol quanta m^−2^ s^−1^ of actinic light (AL) applied to dark-adapted leaves. The ΦPSII was determined using the Φ PSII = (F’m − Fs)/F’m equation, where F’m is the maximum fluorescence obtained with a light-saturating pulse and Fs is the steady-state fluorescence in the light. The Electron Transport Rate (ETR) was calculated with the ETR = Φ_PSII_*PPFD* α* β equation, where PPFD is the photosynthetic photon flux density, α is the leaf absorptance, β is the distribution of absorbed energy between the two photosystems (assumed to be 0.5) and Φ_PSII_ isthe quantum efficiency of the photosystem II. The non-photochemical quenching was calculated with the NPQ = (Fm − Fm′)/Fm equation. The Chlorophyll index was obtained using a chlorophyll-meter (atLEAF, Wilmington, DE, USA). All chlorophyll traits were measured using the second fully expanded leaf from the apical meristem.

### Quantitative Real-Time PCR (RT-qPCR)

Total RNA was extracted from leaves of teak and tobacco transgenic lines using the TRIzol reagent (Thermo Fisher, Waltham, MA, USA) to examine the *TgNAC01* gene expression. Total RNA was quantified using a NanoDrop 2000 spectrophotometer (Thermo Scientific, USA) and its integrity was examined by electrophoresis. The RNA was treated with DNAse I (Promega, USA) and cDNA was synthesized using the SuperScript™ III First-Strand Synthesis System for RT-qPCR (Invitrogen, Waltham, MA, USA), according to the manufacturer’s instructions. Quantitative real-time PCR (RT-qPCR) reactions were carried out using a Platinum Sybr Green Supermix (Invitrogen, Waltham, MA, USA) and run in an ABI 7500 qPCR thermocycler (Applied Biosystems, Waltham, MA, USA). Expression data were normalized using the 2-ΔCt method. The constitutive *Elongation Factor-1 alpha* (*EF-1 alpha*) housekeeping gene was used as internal control for tobacco (Schmidt & Delaney, 2010) and teak (Galeano et al., 2014). Primers were designed using the primer3 software (https://bioinfo.ut.ee/primer3-0.4.0/) (Table S1). We used three biological replicates and two technical repetitions for each transgenic line.

### Statistical analyses

The experiment was conducted in a completely randomized design with three replicates. Differences between means for the physiological parameters were assessed through ANOVA using a univariate general linear model and the standard error of the difference between means (SED) and the post hoc-Tukey HSD test with a *p*-value (*p* < 0.01) for mean comparisons. The gene expression (RT-qPCR) data were subjected to statistical analysis using the Student’s t test. The salt stress assay was analyzed with the y = µ+ Pb + Qc +Sd + *ɛ* model, where y is the vector of each physiological trait, µis the constant of the general mean for each trait, b is the vector for the transgenic lines and WT effect, c is the vector of NaCl concentration effect, d is the vector of interaction between genotypes (transgenic lines and WT) and salt treatment, and *ɛ* is the vector of error effect with *ɛ*N(O, *σ*). Capital letters represent the incidence matrices for each of the effects. Differences between means were assessed through an ANOVA, using a univariate general linear model, and the post hoc-Tukey HSD test at a significance level of *P* < 0.05 for the mean comparisons.

## Results

### Phylogeny and evolution of TgNAC01

**Figure 1:**
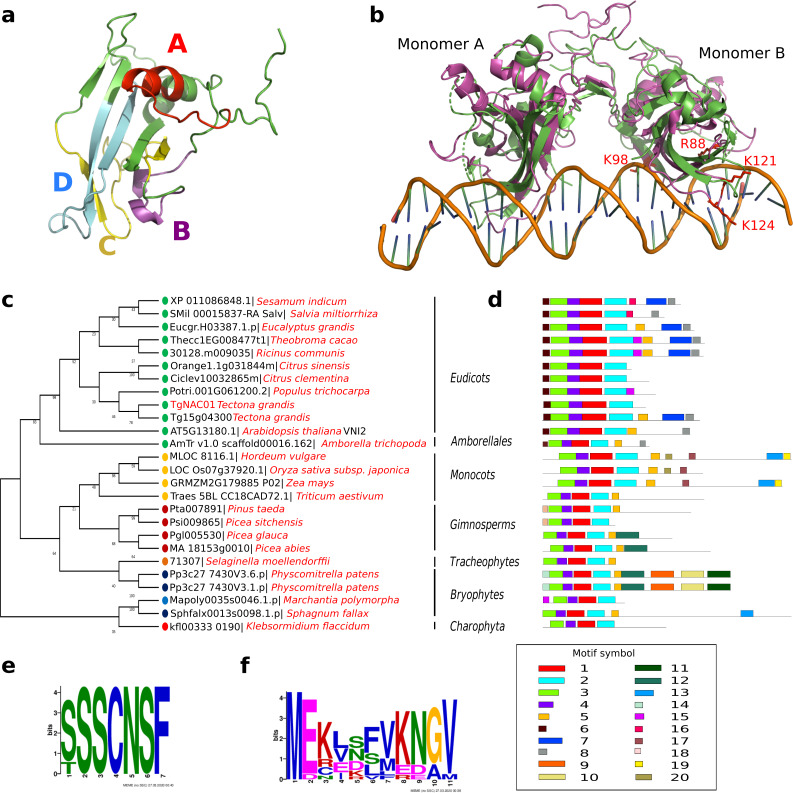
TgNAC01 protein structure prediction, evolutionary relationship and multiple alignment of VNI2-homologous proteins from different species. (A) TgNAC01 protein folding prediction with NAC sub-domains in monomer form. (B) TgNAC01 dimer prediction form (magenta) aligned to ANAC019 protein (RCSB:3SWP) (green). (C) Molecular evolution of TgNAC01 (VNI2 homologous) compared with 22 species (25 proteins). The phylogenetic tree was constructed using the Likelihood method, Pearson correction and 1,000 bootstraps using Mega 7.0. (D) Structure motifs of VNI2 proteins. (E) Logo PEST motif. (F) Logo corresponding to the most conserved motifs of the VNI2 homologous protein in eudicots.

*TgNAC01* consists of a 492 nucleotide coding region, which encodes a protein of 164 amino acid residues, and a typical NAC domain divided into four sub-domains (A, B, C and D) (Ooka et al., 2003) (Fig. 1A). These canonical sub-domains form structures in 5 α-helix and 2 β-folded sheets highly similar to those formed by the ANAC19 dimer and VNI2 monomer (Fig. 1A, Fig. S1). In addition, the TgNAC01 protein appears to have the ability to form dimers through dimer interfaces in the N-terminal region, specifically in the NAC-A subdomain (Fig. 1B). The monomer subdomain B, which is responsible for DNA-binding, is not involved in dimer structures. The teak NAC phylogenetic tree allocated TgNAC01 to the NAC-N group of the teak NAC protein family (Hurtado et al., 2020) sharing the same taxa with Tg15g04300.t1 (Fig. S2). Multiple sequence (Clustal) analysis of TgNAC01 protein revealed 63% amino acid sequence similarity to the Tg15g04300.t1 teak protein. However, the N-Terminal portion of the TgNAC01 and Tg15g04300.t1 proteins shared 96% amino acid sequence identity (158 of 164 amino acids) (Fig. S3). The unrooted phylogenetic tree grouped TgNAC01 with Potri.0001G061200.2 of poplar and Eucgr.H03387.1.p of *Eucalyptus*. Furthermore, the phylogenetic tree identified the AtVNI2 protein as the most recent common ancestor in the eudicots monophyletic group. Besides, the kfl003330190 protein of *klebsormidium flaccidum* (charophyte green algae) and the TgNAC01 protein were clustered in a same paraphyletic group (Fig. 1C). The Tg15g04300.t1 is the paralogous protein of TgNAC01 (Fig. 1C). The TgNAC01 homologous protein motifs showed four sub domains located in the N-terminal region, which are evolutionarily conserved and characteristic of all analyzed species. The C-terminal of the protein is not conserved (Fig. 1D). The Tg15g04300.t1 and VNI2 proteins contain regions rich in proline (P), glutamic acid (E), serine (S), and threonine (T), which are defined as PEST motifs (Fig. 1E). The TgNAC01 protein does not contain the PEST motif suggesting that its function is limited to the NAC domain that regulates transcription. In fact, a highly accurate prediction of TgNAC01 folding alignment with VNI2 revealed that they share a structurally well-defined region in the NAC domain whereas the remaining amino acids form a loop structure. Curiously, the PEST motif is located in the region that form loops (Fig. S1). In addition, the [MEKVSFVKNGV] motif located in the N-terminal region has been found only in eudicots and *Amborella trichopoda* (Fig. 1F).

### *TgNAC01* gene expression patterns in response to abiotic stresses and exogenous phytohormone treatments

**Figure 2:**
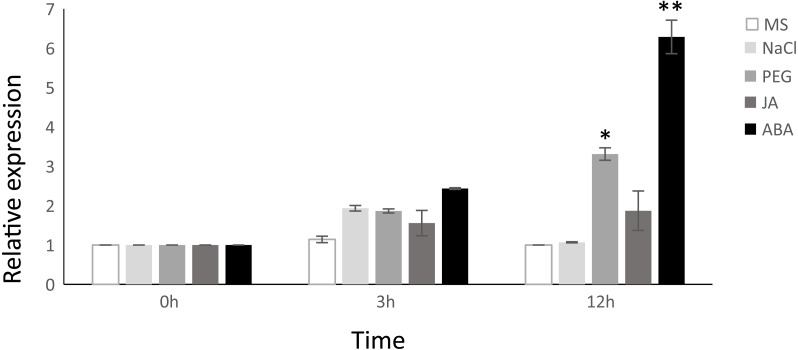
Relative gene expression of *TgNAC01* in response to abiotic stresses and phytohormones assessed through qRT-PCR. Total RNA was isolated from leaves of 60-day-old teak seedlings collected after exposure to 100 µM MeJA, 100 µM ABA, 150 mM NaCl and 20% PEG. Teak seedlings incubated in MS media were used as controls. The constitutive Tg*EF1* α (*Elongation Factor-1 alpha*) housekeeping gene was used as internal control. Values correspond to the means ± standard error of the mean of three biological replicates, * *P* < 0.05 and ** *P* < 0.01.

Considering that the VNI2 induction is ABA-dependent (Yang et al., 2011), we proposed a hypothesis that TgNAC01 protein, a close VNI2 homologous in teak, could have a preserved function. In fact, the highest relative expression was found in plants induced by ABA at 3 and 12 h of treatment. In addition, we tested the response of *TgNAC01* expression in 60-day-old *in vitro* teak seedlings in culture medium supplemented with NaCl, PEG or MeJA (Fig. 2). Gene expression profiles were similar in plants treated with PEG, ABA and MeJA (Fig. 2). Although there was a small rise of *TgNAC01* transcription levels at hour 3 in all treatments, they strongly increased in transgenic plants at hour 12 under PEG and ABA in comparison to the control plants (Fig. 2). These results indicated that abscisic acid induces *TgNAC01* expression, which continues increasing over time during the 12-hour exposure to this hormone.

### Subcellular localization of TgNAC01 protein

**Figure 3:**
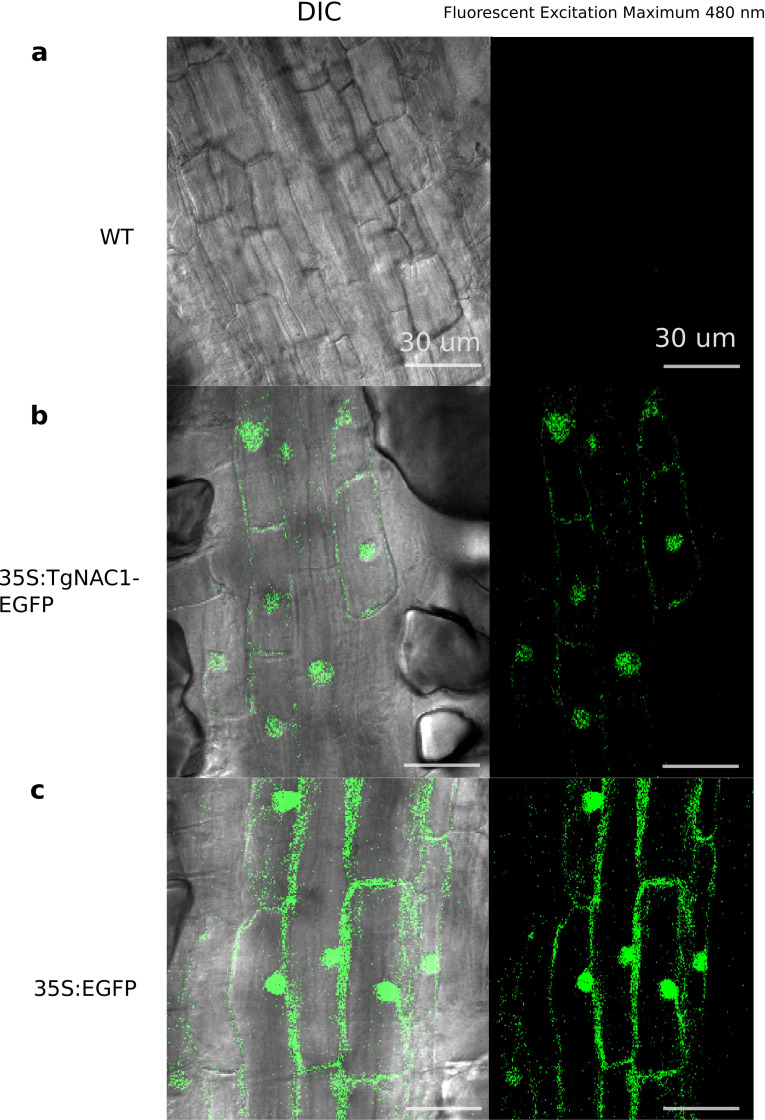
Subcellular localization of the TgNAC01:GFP protein in roots of transgenic tobacco seedlings. Cells of root elongation zone of seven-day-old seedlings of tobacco lines expressing 35S:TgNAC01-EGFP or 35S:EGFP, and the wild type (WT), were observed under microscopy of differential interference contrast(DIC) and fluorescence excitation. (A) WT roots without fluorescence. (B) The TgNAC01-1 transgenic line showing GFP fluorescence in the nucleus. (C) 35S:EGFP showing GFP fluorescence in the nucleus and cytoplasm.

As represented in Fig. 3B, no fluorescence was observed in WT tobacco root samples (Fig. 3A), whereas the stable 35S:TgNAC01-EGFP tobacco transgenic plants, confirmed by PCR (Fig. S8), clearly showed high intensity of EGFP fluorescence in the nucleus. This result was confirmed by Differential Interference Contrast (DIC). Furthermore, a computational prediction confirmed the TgNAC01 protein localization in the nucleus since this protein lacks the NAC WITH TRANSMEMBRANE MOTIF1 (NTM1) (Fig. S7). Moreover, the 35S:EGFP fluorescence was observed in both nucleus and cytoplasm (Fig. 3C), thus this result contrasts with the subcellular fluorescence localization of 35S:TgNAC01-EGFP limited to the nucleus. Also, through a computational prediction, we found that TgNAC01 is localized in the nucleus (Fig. S6, Tables S2–S3).

### Characterization of TgNAC01 transgenic tobacco plants

**Figure 4:**
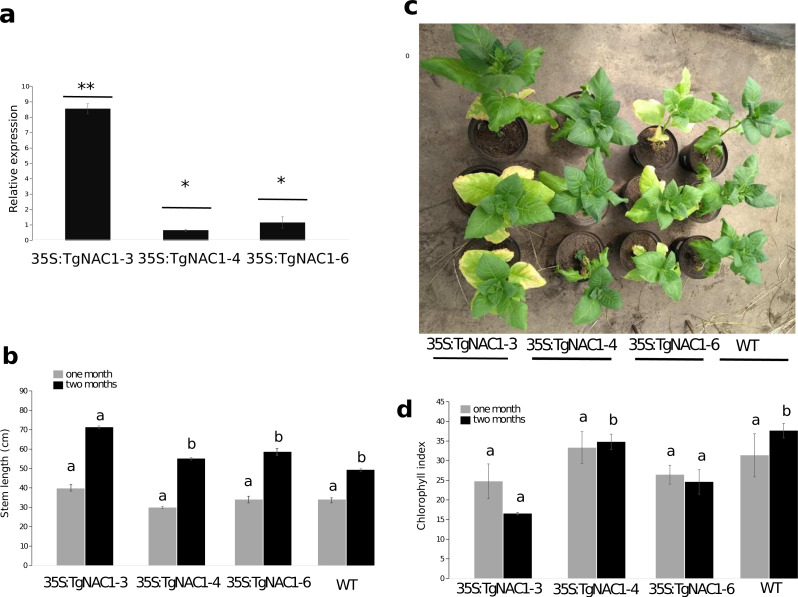
Phenotype, growth and leaf senescence of 35S:TgNAC01-3, 35S:TgNAC01-4, 35S:TgNAC01-6 tobacco transgenic lines and WT plants 60 days after germination. (A) Relative expression of TgNAC01 in leaves of transgenic lines. Means correspond to three measurements. The standard error of the mean is shown as bars. Asterisks indicate differences between means for relative expression significant at *p* > 0.01. (B) Growth of 35S:TgNAC01 lines and WT measured at days 30 and 60 after germination. (C) TgNAC01 transgenic lines of tobacco and WT plants 60 days after germination. (D) Chlorophyll index measured at days 30 and 60 after germination. Values represent the means ±standard error of the mean calculated using three replicates. Differences between means were considered to be significant at *p* < 0.01 by Tukey multiple comparison tests, calculated for one and two months separately. Lowercase letters represent the differences among transgenic lines and WT plants.

Among eight independent 35S:TgNAC01 transgenic lines, the 35S:TgNAC01-3, 35S:TgNAC01-4, and 35S:TgNAC01-6 lines were identified as homozygous for *TgNAC01* in the T2 generation under kanamycin selection (100 mg L^−1^). In the three lines the transgene insert was confirmed by PCR (Fig. S4) and RT-qPCR (Fig. 4A). In comparison to WT plants, roots were significantly shorter in these three transgenic lines (Fig. S5). Notably, the 35S:TgNAC01-3 roots were longer and with significant higher relative expression compared to the two other transgenic lines. In previous studies, VNI2 (TgNAC01 homologous) was described as a negative regulator of the protoxylem development and this function is possibly associated with the pPEST motif post translational control of the VND7 function (Yamaguchi et al., 2010). Surprisingly, TgNAC01 does not have the PEST motif. Further studies using the Tg15g04300 paralogue containing the PEST motif will improve the understanding of the role of this motif in root development (Fig. 1).

Stem length differences were observed in plants at one month of growth under optimal conditions in the greenhouse. Stem length of 35S:TgNAC01-3 plants were 30% longer compared to WT plants (Fig. 4B, Fig. S5B). As for 35S:TgNAC01-4 and 35S:TgNAC01-6 lines, there were no significant statistical differences between them, even though their stems were, respectively, 20 and 25% longer than WT plants (Figs. 4A–4B). Length differences in these teak plants could be due to higher *TgNAC01* gene expression in the stem secondary xylem (50-fold) and branch (20-fold) compared to leaves and roots (Camel, Galeano & Carrer, 2017). Senescence of basal leaves was observed after one month of growth in the 35S:TgNAC01-3 transgenic line (Fig. 4C). Besides, chlorophyll content in this transgenic line was significantly lower after two months of growth compared to the other lines and control plants (Figs. 4C–4D). Considering the hypothesis that *TgNAC01* overexpression in tobacco plants accelerates senescence, it may also affect other physiological responses in plants under stress conditions.

### Role of *TgNAC01* under salt stress

**Figure 5:**
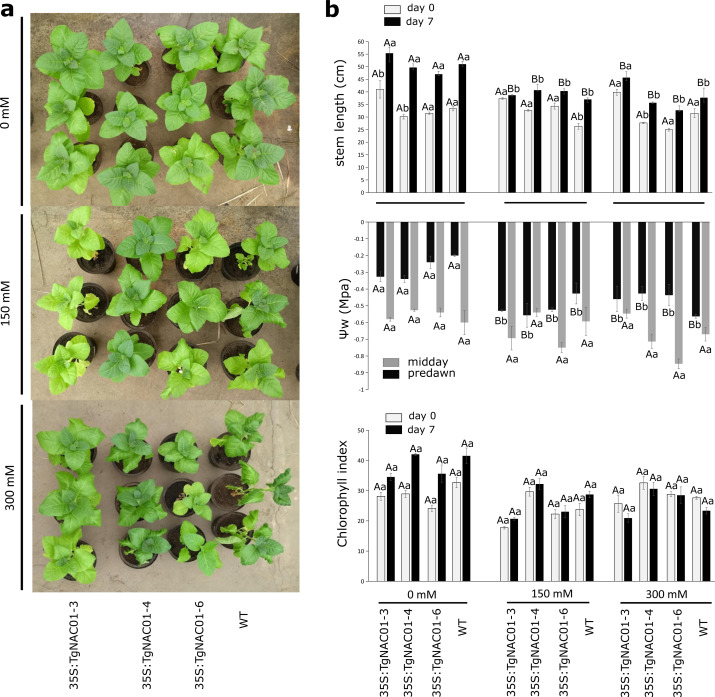
Phenotype, leaf water potential and chlorophyll index of 35S:TgNAC01-3 35S:TgNAC01-4, 35S:TgNAC01-6 tobacco transgenic lines and WT under salt stress. (A) Phenotype of 35S:TgNAC01 lines and WT after seven days under three NaCl concentrations. (B) Stem length (cm) measured at day 0 and after seven days on salt stress, leaf water potential (MPa) measured after seven days of salt stress and chlorophyll index measured at day 0 and day 7 of salt stress. Values represent the standard error calculated using three replicates. Differences between means were considered to be significant at *P* < 0.01 by Tukey multiple comparison tests, calculated for days 0 and 7 separately. Capital letters represent the difference related to the effect of the treatment (salt concentration) and lowercase letters represent the differences among transgenic lines and WT plants.

Up to the seventh day of salinity stress, the 150 and 300 mM NaCl treatments decreased plant growth of WT and transgenic lines, except for line 35S:TgNAC01-3 (Fig. 5A). These transgenic plants showed higher growth even at day 7 under 300 mM NaCl stress compared to the other transgenic lines and WT plants (Fig. 5B). Leaf water potential (*ψ*_w_) was measured in the predawn and midday at day 7 of NaCl stress. Basal potential (predawn) decreased (more negative values) in all transgenic lines compared to control plants under salt stress (Fig. 5B). Minimum water potential values in predawn occurred in WT plants under 300 mM salinity stress. However, at midday no significant differences in water potential parameters were observed between WT plants and all transgenic lines under salt stress (Fig. 5B). Besides, there were no significant statistical differences in chlorophyll contents (Fig. 5B). However, chlorosis was observed in baseline leaves of 35S:TgNAC01-4 and WT plants treated with 300 mM NaCl (Fig. S9).

### Gas exchange parameters of tobacco transgenic lines overexpressing *TgNAC01* under salt stress

**Figure 6:**
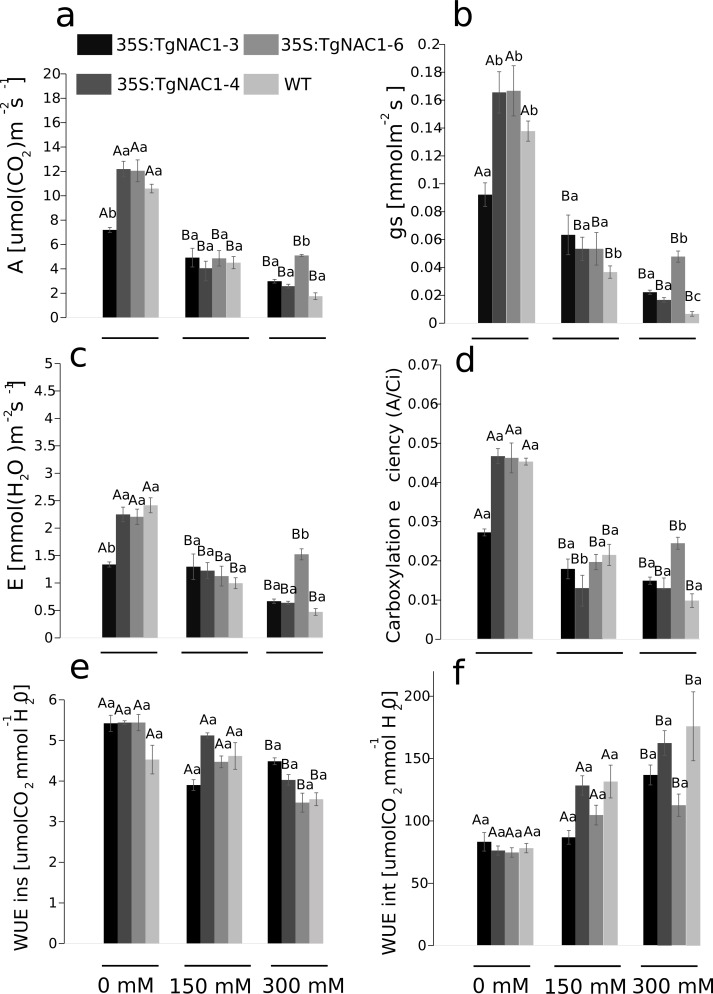
Gas exchange parameters in tobacco transgenic lines and WT plants at day 7 of salt stress. (A) Photosynthetic rate (A). (B) Stomatal conductance (gs). (C) Transpiration rate (E). (D) Carboxylation efficient (A/intercellular CO2 concentration). (E) Instantaneous water use efficiency (WUEins). (F) Intrinsic water use efficiency (WUEint). Values represent the means ± standard error of the mean calculated using three replicates. Differences between means were considered to be significant at *P* < 0.01 using the Tukey multiple comparison tests. Capital letters represent the difference due to the effect of the treatments (salt concentration) and lowercase letters represent the difference among transgenic lines and WT plants.

Photosynthesis (A), stomatal conductance (gs), transpiration rate (E) and carboxylation efficiency (A/Ci) decreased proportionally from 0 to 300 mM of salt concentration in all plants (Figs. 6A–6D). Contrastingly, photosynthetic rates were increased at day 7 of 300 mM NaCl treatment in transgenic lines compared to WT plants (Fig. 6A). The 35S:TgNAC01-3 transgenic line showed the lowest values of gas exchange when no salt stress was applied (Fig. 6B). In day 7 of the 300 mM NaCl stress, the most significant increase of A, gs, E and A/Ci occurred in plants of the 35S:TgNAC01-6 line in comparison to the other transgenic lines and WT plants (Figs. 6A–6D). The 35S:TgNAC01-6 line started with a gas exchange response lower than the other transgenic lines and control plants under 150 mM NaCl (4.41 µmol (CO_2_) m^−2^s^−1^ of A, 0.06 mmol m^−2^s^−1^ of gs and 1.34 µmol (H_2_O) m^−2^s^−1^ of E). Surprisingly, in the seventh day under 300 mM NaCl stress the 35S:TgNAC01-6 plants responded with gas exchange values higher (5.24 µmol (CO_2_) m^−2^s^−1^ of A, 0.06 mmol m^−2^s^−1^of gs, 1.5 µmol (H_2_O) m^−2^s^−1^ of E) than control plants (1.75 µmol (CO_2_) m^−2^s^−1^ of A, 0.01 mmol m^−2^s^−1^ of gs, 0.42 µmol (H_2_O) m^−2^s^−1^ of E) (Figs. 6A–6D). While instantaneous WUE values of transgenic lines and WT plants were increased in all conditions, with and without salt stress (Fig. 6E), significant increase of intrinsic WUE values in WT and transgenic lines was determined by 150 and 300 mM NaCl treatments (Fig. 6F).

### Chlorophyll fluorescence of tobacco transgenic lines overexpressing *TgNAC01* under salt stress

**Figure 7:**
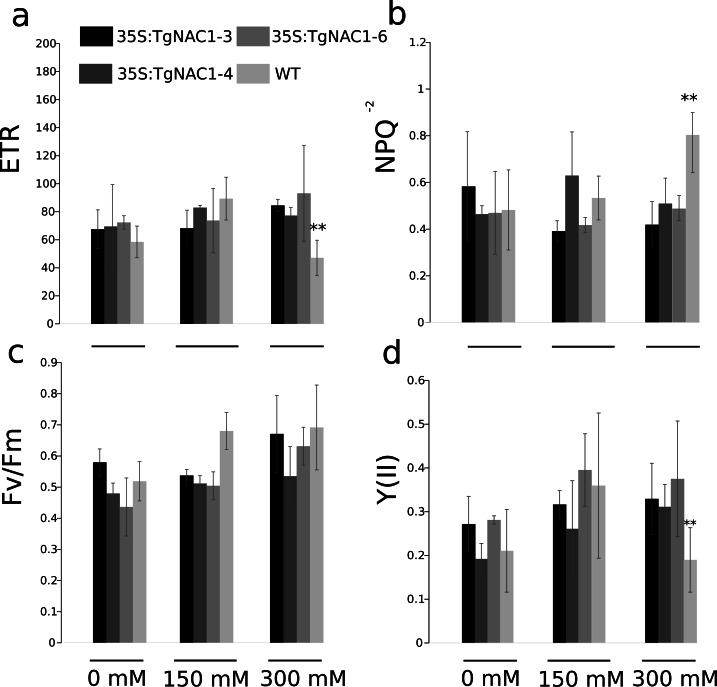
Chlorophyll fluorescence parameters in tobacco transgenic lines and WT plants under salt stress. (A) Electron transport rate (ETR). (B) Non-photochemical quenching (NPQ). (C) Maximum quantum yield of PSII photochemistry. (D) Effective photochemical quantum yield of PSII (YII). Values are the means ± standard error of the mean calculated using three replicates. Differences between means were considered to be significant at *P* < 0.01 using Tukey multiple comparison tests. Asterisks represent the statistically significant difference *P* < 0.01.

Statistically significant differences in chlorophyll fluorescence parameters were observed during the stress period between WT plants and transgenic lines under 300 mM NaCl (Fig. 7). Even though the electron transport rate (ETR) showed significant differences between control plants and transgenic lines treated with 300 mM NaCl (Fig. 7A), all plants showed a slight and general reduction in the ETR at seven days of salt stress with the lowest values at 300 mM NaCl, particularly in the WT plants (Fig. 7A). In accordance with the ETR results, the non-photochemical quenching (NPQ) values were significantly different between transgenic lines and WT plants exposed to 300 mM NaCl (Fig. 7B). Specifically, the WT plants showed a significant NPQ increase (Fig. 7B). Differences in maximum quantum efficiency of PSII photochemistry (Fv/Fm) between treatments were not statistically significant, but there was a general slight increase under 300 mM NaCl (Fig. 7C). PSII operating efficiency [Y(II)] increased in all conditions and genotypes (Fig. 7D), however it was significantly reduced in WT plants in comparison to the transgenic lines (Fig. 7D). As for ETR and Y(II)n, the response of WT plants under saline stress in general was lower compared to the transgenic lines (Figs. 7A–7D).

## Discussion

### TgNAC01 protein evolution and localization

The NAC protein family has been described in a wide range of land plants. Thus far, NAC TF genes have not been found in single algae, such as the rhodophyta *Cyanodioschyzon merolae*, the glaucophytes *Cyanophora paradoxa* and *Glaucocystis nostochinearum*, and the chlorophytes *Ostreococcus tauri*, *Chlorella vulgaris*, *Chlamydomonas reinhardtii* and *Volvox carteri* (Maugarny-Calès et al., 2016). Nonetheless, homologous NAC proteins were found in groups of streptophyte pluricellular green algae and it was suggested that NAC TFs appeared before the emergence of land plants during the Klebsormidiales divergence (Maugarny-Calès et al., 2016). We consistently found that the closest TgNAC01 ancestors were the kfl003330190 gene of *Klebsormidium flaccidium*, a filamentous green alga, and VNI homologous proteins from Amborellales, a plant order thought to represent the earliest diverging branch among living members of angiosperms (Fig. 1C). The TgNAC01 protein belongs to the NAC-N group in the NAC protein family (Hurtado et al., 2020), which presumably originated from gene duplication events. It shares a near evolutionary origin with the *AT2G33480.1* (*ANAC83-AtVNI2*) gene of Arabidopsis, as previously described (Hurtado et al., 2020). In general, the N-terminal portion of NAC family members is conserved among different plant species and shares motifs with eudicots VNI homologous proteins (Li et al., 2012). Protein diversity has been traditionally associated with improved or diversified molecular function, which is directly related to protein structure. We identified a conserved region between TgNAC01 and the homologous AtVNI2 protein, particularly at the first five motifs of the alignment (Figs. 1D–1F). Furthermore, the [MEKVSFVKNGV] motif located in the N-terminal region has been found only in eudicots and *Amborella trichopoda*, thus suggesting that the AtVNI2 homologous protein appeared during the Amborellales divergence (Figs. 1D–1F). Nevertheless, the TgNAC01 protein lacks the PEST proteolysis target motif [TTDLNLLPSSPSSD] (Figs. 1D–1E), which exists in the AtVNI2 C-terminal region. This motif is known to promote rapid protein degradation (García-Alai et al., 2006) and is suggested to be responsible for the degradation of the VND7 negative regulator (Yamaguchi et al., 2010). Interestingly, according to our predictions, the PEST motif is located in a loop (PEST-loop) (Fig. S1). The lack of the PEST motif in TgNAC01 could mean that this protein might not be a negative regulator for the xylem differentiation. Our predictive analysis found that TgNAC01 possibly forms a dimeric protein, which is expected since this form is prevalent in transcription factors. However, further analyses of the molecular mechanism of TgNAC01 are needed for better understanding this dimerization assumption. The dimeric interface of TgNAC01 is located in the N-terminal region specifically in the NAC-A sub-domain (Fig. 1B), which is similar to folding described in other NAC proteins (Ernst et al., 2004). Amino acid residues R88, K96, K123 and K129 have been described in β-4 and β-5 loops of the chain-B in ANAC019, whose structure and folding were elucidated by X-ray diffraction. These Arginine and Lysine amino acids are known to physically interact with nucleotide sequences (Welner et al., 2012; Welner et al., 2016). The TgNAC01 dimer has similar conserved positions (R88, K98, K121 and K124) (Fig. 1B). Therefore, it is conceivable to hypothesize that these sequences and folding are conserved among NAC transcription factors. Furthermore, computational predictions and absence of NTM1 indicated that the TgNAC01 protein is located in the nucleus. Besides, microscopy analysis of TgNAC01 heterologous expression in tobacco further confirmed this protein in the nucleus (Fig. 3B). The NTM1 region was reported in 13 and six NAC proteins of Arabidopsis and rice, respectively (Kim et al., 2007). Analysis of stem-differentiating xylem of *P. trichocarpa* found six VNDs being exclusively overexpressed in the nucleus (Lin et al., 2017). Moreover, Arabidopsis protoplasts showed overexpression of the *AtVNI2* gene in the nucleus (Yang et al., 2011).

### *TgNAC01* has a role in growth and regulation of senescence

Xylem cell differentiation consists of four biological processes: secondary cell wall (SCW) deposition, programmed cell death, autolysis and lignification (Zhong, Cui & Ye, 2019). Overexpression of *TgNAC01* could affect SCW deposition since this transcription factor has been demonstrated to be a switch with higher expression in teak secondary xylem in comparison with other tissues (Camel, Galeano & Carrer, 2017). Consequently, it may increase stem length by affecting periclinal cell division during the radial stem expansion (Tonn & Greb, 2017). In our study, high TgNAC01 expression in 35S:TgNAC01-3 tobacco plants accelerated growth and foliar senescence. Actually, 35S:TgNAC01-3 plants developed stems 30% longer than WT plants (Figs. 4A–4B). It is known that the *TgNAC01* gene is highly expressed in lignified tissues and was reported to be 50-fold higher in stem secondary xylem tissues compared to leaves and roots (Camel, Galeano & Carrer, 2017). Our data provide further evidence that overexpression of this TF gene increases plant height possibly in response to increased lignin. Overexpression of *SND2* in Arabidopsis increased cell wall thickening in xylem fibers with a consequent increase in plant height (Zhong et al., 2008). In other plant species, NAC TFs were also found to regulate cell wall thickening during fiber development, such as the NAC15 TF in *Populus* (Zhong, Lee & Ye, 2010a; Zhao et al., 2014). The *PtrNAC15* gene overexpressed in transgenic tobacco plants played a significant role in wood formation (Yao et al., 2020). In agreement with our results, the *NAC13* gene of poplar (*Populus alba* × *P. glandulosa*) overexpressed in transgenic tobacco plants showed a positive regulation under salt stress resulting in a higher plant growth compared to control plants (Zhang et al., 2019). Additionally, NAC transcription factors have been reported to play a crucial role in regulation of leaf senescence (Kim, Nam & Lim, 2016; Li et al., 2018). Usually, NAC regulation of leaf senescence is associated with changes in gene expression patterns, structure and metabolism, including a loss of photosynthetic activities and an increase in hydrolysis of proteins, membrane lipids, and nucleic acids (Woo et al., 2013). In Arabidopsis, leaf senescence was induced by overexpression of *AtNAP/ANAC029* and *ORE1/ANAC092 NAC*-related transcription factors (Guo & Gan, 2006). Expression of *AtVNI2* was higher in senescent leaves compared to healthy ones (Yang et al., 2011). Most likely, acceleration of leaf senescence in transgenic tobacco plants overexpressing *TgNAC01* was triggered by ABA-signaling in response to the saline stress (Fig. 5). A strong association of premature leaf senescence with NAC TFs and ABA signaling has been described in several species (Li et al., 2018). Molecular mechanisms regulating this ABA signaling have been under investigation (Kim, Nam & Lim, 2016; Kim et al., 2018)

### *TgNAC01* confers salt stress tolerance

NAC family members have been reported as essential in the regulation of stress tolerance (Nuruzzaman, Sharoni & Kikuchi, 2013; Hoang et al., 2020). The Arabidopsis NAC-related genes *ANAC019*, *ANAC055* and *ANAC072* were identified as stress regulators since they were induced and responded with enhanced tolerance under multiple abiotic stresses and exogenous hormones, such as drought, high salinity, ABA and MeJA (Jensen et al., 2010; Li et al., 2012). In our study, *TgNAC01* gene expression responded to NaCl, PEG, ABA and MeJA stress treatments and phytohormone-signaling molecules (Figs. 2A–2B). The highest increase in *TgNAC01* expression was promoted by ABA (Fig. 2B) and indicated that this TF gene may be involved in stress tolerance in teak. The higher leaf water potential in predawn of transgenic tobacco plants overexpressing this NAC TF, in comparison with WT plants (Fig. 5B), suggested that these plants under salt stress could counteract excessive loss of water, as previously described (He et al., 2019). Furthermore, it is plausible that the increase of midday leaf water potential was due to stomatal closing and no transpiration (Fig. 5B). As widely reported, salt accumulation in roots may cause a decrease in osmotic potential and, consequently, a decrease in leaf water potential (Franco et al., 2011). It is conceivable that high levels of TgNAC01 protein in 35S:TgNAC01-3 plants were transducing the ABA signal at greater intensity and, consequently, leading to accelerated foliar senescence up to 60 days after germination. Actually, reduction of gas exchange effectiveness was detected earlier, in 30-day-old plants, possibly due to stomatal closure by increased ABA-transduction. These 35S:TgNAC01-3 plants showed the least negative leaf water potential value compared to 35S:TgNAC01-4, 35S:TgNAC01-6 and WT plants (Fig. 5B). *TgNAC01* overexpression increased tolerance to water loss in transgenic tobacco plants (Figs. 6C–6F). Similar results on water loss reduction were observed in *ThNAC7* from *Tamarix hispida* overexpressed in Arabidopsis (He et al., 2019). *AtVNI2* overexpression under high salinity induced transcriptional activation of *COR/RD* genes and promoted tolerance to saline stress (Yang et al., 2011). Likewise, overexpression of *CarNAC3* and *CarNAC6* transcription factors from *Cicer arietinum* in poplar transgenic lines conferred tolerance to high salt stress, whereas WT plants presented withered leaves and stopped growing (Movahedi et al., 2015). There was a reduction of A, gs, E and A/Ci in *TgNAC01* transgenic lines at day 7 of salt stress (Fig. 6). The decrease in carboxylation efficiency rate indicated that salt stress affects photosynthesis possibly due to a metabolic limitation associated with a decrease in the rubisco carboxylase activity (Najar et al., 2019). Previous studies have shown that several NAC transcription factors regulate stomatal movements *via* ABA signaling mediated by PEG-induced drought response (Bielsa et al., 2018). Data on TgNAC01 overexpression suggested that this transcription factor may improve water use efficiency (Figs. 5–6). The highest photosynthetic rate (A), stomatal conductance (gs) and transpiration (E) was observed in the 35S:TgNAC01-4 line under 300 mM NaCl (Figs. 6A–6D). However, 35S:TgNAC01-6 transgenic plants showed a better response under salt stress regarding A, gs, E, and carboxylation efficiency (A/Ci), in spite of premature leaf senescence, in comparison with 35S:TgNAC01-4 and WT plants (Figs. 5–6). This result suggests that increased *TgNAC01* transcript levels induce a greater response to ABA. Interestingly, the highest tolerance to salt stress was observed in 35S:TgNAC01-6 transgenic plants indicating a dose effect of gene expression. The data suggest that *TgNAC01* could be investigated as a novel candidate gene for biotechnological improvement of teak. Similar results were described for *AtJUB1*, a NAC gene of Arabidopsis that is related to the TgNAC01 teak. AtJUB1 was overexpressed in tomato transgenic plants under high salinity, which were more efficient to maintain biomass and physiological parameters in comparison with WT plants (Alshareef et al., 2019). In addition, transgenic tobacco lines overexpressing *TsNAC1* from *Thellungiella alophile* showed reduced growth and improved survival after exposure to drought and high-salt stress compared to control plants (Liu et al., 2018). Contrastingly, *PeNAC045* overexpression in *Populus* showed lower A, gs and E compared to WT plants, with additional reduction in drought and salt tolerance (Lu et al., 2018). Furthermore, we observed that ETR and effective photochemical quantum yield of PSII increased in the transgenic lines (Fig. 7). It is known that the electron transport rate increases proportionally with carbon fixation and that stress conditions can drastically decrease these two parameters (Mishra, 2018). Therefore, the observed low photosynthesis values and electron transport rate are indeed expected in WT plants under salt stress but not in *TgNAC01* transgenic lines (Fig. 6A). Moreover, non-photochemical quenching (NPQ) values were higher in WT plants exposed to 300 mM NaCl compared to transgenic lines (Fig. 7). NPQ represents a heat dissipation, which is a plant mechanism of photo-protection, state transition and photo-inhibition. NPQ increases under stress conditions, such as high light intensity or photoinhibition, salinity, heavy metal toxicity, drought or chilling (Mishra, 2018). Transgenic plants maintained relatively the same amount of NPQ in the different salt concentrations compared to WT plants (Fig. 7B), which could mean that the *TgNAC01* gene contributes to the regulation of heat dissipation and photo-protection mechanisms.

### Schematic Model for the *TgNAC01* relevance to senescence and abiotic stress

**Figure 8:**
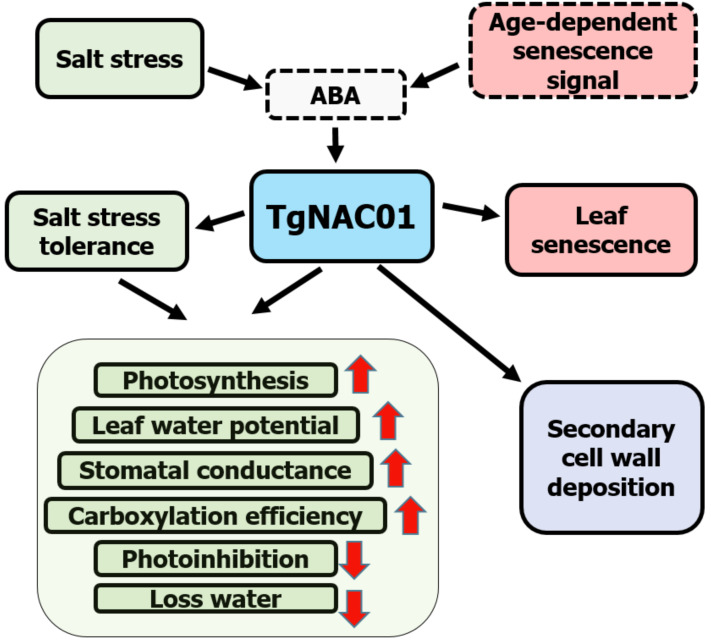
Schematic model for the roles of the *TgNAC01* gene in teak. Green squares depict the salt stress tolerance promoted by *TgNAC01* and the physiological response in teak. Red squares indicate leaf senescence induced by *TgNAC01*. The purple square indicates the role of *TgNAC01* in secondary cell wall deposition, as previously reported (Camel, Galeano & Carrer, 2017). Squares with solid lines represent teak processes previously studied and currently in research. Squares with dash lines refer to functions previously reported in other plant species and requiring further elucidation in teak (Yang et al., 2011).

Data demonstrated that 35S:TgNAC01-6 transgenic plants are tolerant to salt stress, whereas WT tobacco plants were the most affected by salinity treatments (Fig. 6). TgNAC01 regulatory functions described in the present work, in conjunction with data from previous studies in teak and its close homologous of Arabidopsis (Yang et al., 2011; Camel, Galeano & Carrer, 2017), are summarized in a model represented in Fig. 8. The *TgNAC01* gene appears as a bifunctional transcription factor, which acts as a positive regulator in the secondary xylem development, as well as a transcriptional activator of leaf senescence and of salt stress ABA signaling. Data suggested that *TgNAC01* may confer salinity tolerance through an ABA-mediated response (Fig. 8, green squares). Thereby, tobacco plantlets under salt stress undergo different physiological responses mediated by *TgNAC01*, such as improved responses in photosynthesis rate, leaf water potential, stomatal conductance, carboxylation efficiency, and a decrease in photoinhibition and water loss (Fig. 8, green squares). Moreover, *TgNAC01* plays a role in leaf senescence, which is probably triggered by ABA signaling (Fig. 2) and activated by an age-dependent senescence signal (Yang et al., 2011); (Fig. 8, red squares). Finally, *TgNAC01* was proposed to have a role in the deposition and differentiation of the secondary cell wall (Camel, Galeano & Carrer, 2017). Data on the stem growth increase of 35S:TgNAC01-3 transgenic plants support this hypothesis (Fig. 4). A clear example of regulation in these two physiological processes was reported for the NST1 gene, which regulates secondary cell wall formation and lignin deposition in *Arabidopsis* through ABA-mediated phosphorylation (Liu et al., 2021).

## Conclusions

The *TgNAC01* amino acid sequence is conserved among species at its N-terminal portion, specifically in the region that characterizes the NAC family name. The TgNAC01 transcription factor lacks the PEST motif, which exists in its AtVNI2 homologous of Arabidopsis. Therefore, most likely TgNAC01 does not conserve the function of a negative regulator of xylem development. *TgNAC01* may be involved in signaling events that mediate teak response to osmotic stress as indicated by its rapid expression induction in teak seedlings under multiple abiotic stress and phytohormone treatments (PEG, NaCl, ABA and MeJA). Furthermore, predictions of protein folding revealed TgNAC01 structures that are conserved with the VNI2 and ANAC19 homologous proteins, both related to response to osmotic stress. In accordance with its role as a transcription factor, the expression of *TgNAC01* was located exclusively in the cell nucleus of transgenic tobacco plants. In addition, this gene lacks transmembrane motifs. The *TgNAC01* gene is associated with senescence regulation as indicated by premature leaf senescence and reduction of chlorophyll content in the leaf base of 60-day-old transgenic tobacco plants. High *TgNAC01* transcript levels increased response to ABA. Besides, the data indicated a *TgNAC01* dose effect was evidenced by different levels of salt stress tolerance among transgenic lines and wild type plants. Ultimately, our study suggests that *TgNAC01* is a bifunctional transcription factor, acting as a positive regulator of the secondary xylem development and as a transcriptional activator in stress-mediated ABA regulation and leaf senescence. Data suggested *TgNAC01* as a potential novel candidate gene for investigations on biotechnological improvement of stress tolerance in teak.

## Supplemental Information

10.7717/peerj.13039/supp-1 Supplemental Information 1 Folding protein prediction with Alphafold2 (A) Folding alignment protein between TgNAC01 and VNI2 (ANAC083) all in monomer forms. (B) Folding in dimer form of TgNAC01 with probable amino acids responsible for DNA binding.

10.7717/peerj.13039/supp-2 Supplemental Information 2 Phylogenetic tree of teak NAC proteins and TgNAC01 The phylogenetic tree was constructed using the Likelihood method, pearson correction and 1,000 bootstraps using Mega 7.0. Blue letter is Tg15g04300 and red letter is TgNAC01.

10.7717/peerj.13039/supp-3 Supplemental Information 3 CLUSTAL alignment of TgNAC01 and Tg15g04300 proteins of teak Red letter shows amino acid identity between TgNAC01 and Tg15g04300

10.7717/peerj.13039/supp-4 Supplemental Information 4 Plant transgenic confirmation (A) Agarose gel electrophoresis (1%) of the PCR product corresponding to the 35S:TgNAC01 transgenic lines, using a 1kb Plus DNA Ladder (Invitrogen), with 0.9 µg/lane, 0.9% of agarose stained with ethidium bromide. From lane 1–11: tobacco transgenic lines. Lane 11 corresponds to WT plants. Lane (+) corresponds to the plasmidial DNA from *E. coli* carrying the 35S:TgNAC01 construction. Lane (-) corresponds to H_2_O miliQ. (B) Nine-days-old after germination 35S:TgNAC01-3, 35S:TgNAC01-4, 35S:TgNAC01-6, 35S:TgNAC01-9 transgenic lines and WT tobacco seedlings, in a medium selection supplemented with 100 mg L-1 kanamycin

10.7717/peerj.13039/supp-5 Supplemental Information 5 Characterization of *TgNAC01* transgenic lines in tobacco (A) Root length of 35S:TgNAC01 transgenic lines and WT plants seven days after germination. (B) Phenotype of 35S:TgNAC01 lines and WT at day 60 after germination. Means correspond to three measurements. The standard error of the mean is shown as bars. Letters explain differences between means for root length that 99% of confidence.

10.7717/peerj.13039/supp-6 Supplemental Information 6 Subcelullar localization prediction Subcellular localization prediction of TgNAC01 protein using Loctree 3 (https://rostlab.org/services/loctree3).

10.7717/peerj.13039/supp-7 Supplemental Information 7 Transmenbrane predict Transmenbrane predict of TgNAC01 protein using HMMTOP (http://www.enzim.hu/hmmtop/index.php).

10.7717/peerj.13039/supp-8 Supplemental Information 8 Plant transgenic confirmation with EGFP Agarose gel electrophoresis (1%) of the PCR product corresponding to the 35S:TgNAC01-EGFP transgenic lines, using a 1kb Plus DNA Ladder(Invitrogen), with 0.9 µg/lane, 0.9% of agarose stained with ethidium bromide. From Lane 1–7: tobacco transgenic lines. Lane 8 correspond to WT plants. Lane 9 corresponds to the plasmidial DNA E.coli carrying the 35S:TgNAC01 construction. Lane 10 corresponds to H_2_O miliQ.

10.7717/peerj.13039/supp-9 Supplemental Information 9 Effect of saline stress Phenotype of 35S:TgNAC01 transgenic lines and WT after 7 days under 0 and 300 mM of NaCl concentration

10.7717/peerj.13039/supp-10 Supplemental Information 10 List of primers Primers used for the different experiments performed in the study, and for the various genes.

10.7717/peerj.13039/supp-11 Supplemental Information 11 Subcelullar localization Subcellular localization prediction of TgNAC01 protein using CELLO v.2.5 (http://cello.life.nctu.edu.tw/).

10.7717/peerj.13039/supp-12 Supplemental Information 12 Subcellular localization Subcellular localization prediction of TgNAC01 protein using WoLF PSORT (https://wolfpsort.hgc.jp/).

10.7717/peerj.13039/supp-13 Supplemental Information 13 Chlorophyll fluorescence

10.7717/peerj.13039/supp-14 Supplemental Information 14 Gas exchange parameter plants

10.7717/peerj.13039/supp-15 Supplemental Information 15 Table length, water potential and chlorophyll

## Additional Information and Declarations

### Data Availability

The following information was supplied regarding data availability: The raw measurements are available in the Supplementary Files.
