# Supplementary figures and images for: Overexpression of the Tectona grandis TgNAC01 regulates growth, leaf senescence and confer salt stress tolerance in transgenic tobacco plants
Source: PeerJ. 2022 Mar 3;10:e13039. doi: 10.7717/peerj.13039 (PMC8898551; doi:10.7717/peerj.13039)

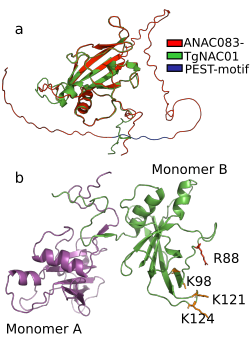

Supplement: Supplemental Information 1 — (A) Folding alignment protein between TgNAC01 and VNI2 (ANAC083) all in monomer forms. (B) Folding in dimer form of TgNAC01 with probable amino acids responsible for DNA binding. [file peerj-10-13039-s001.png]

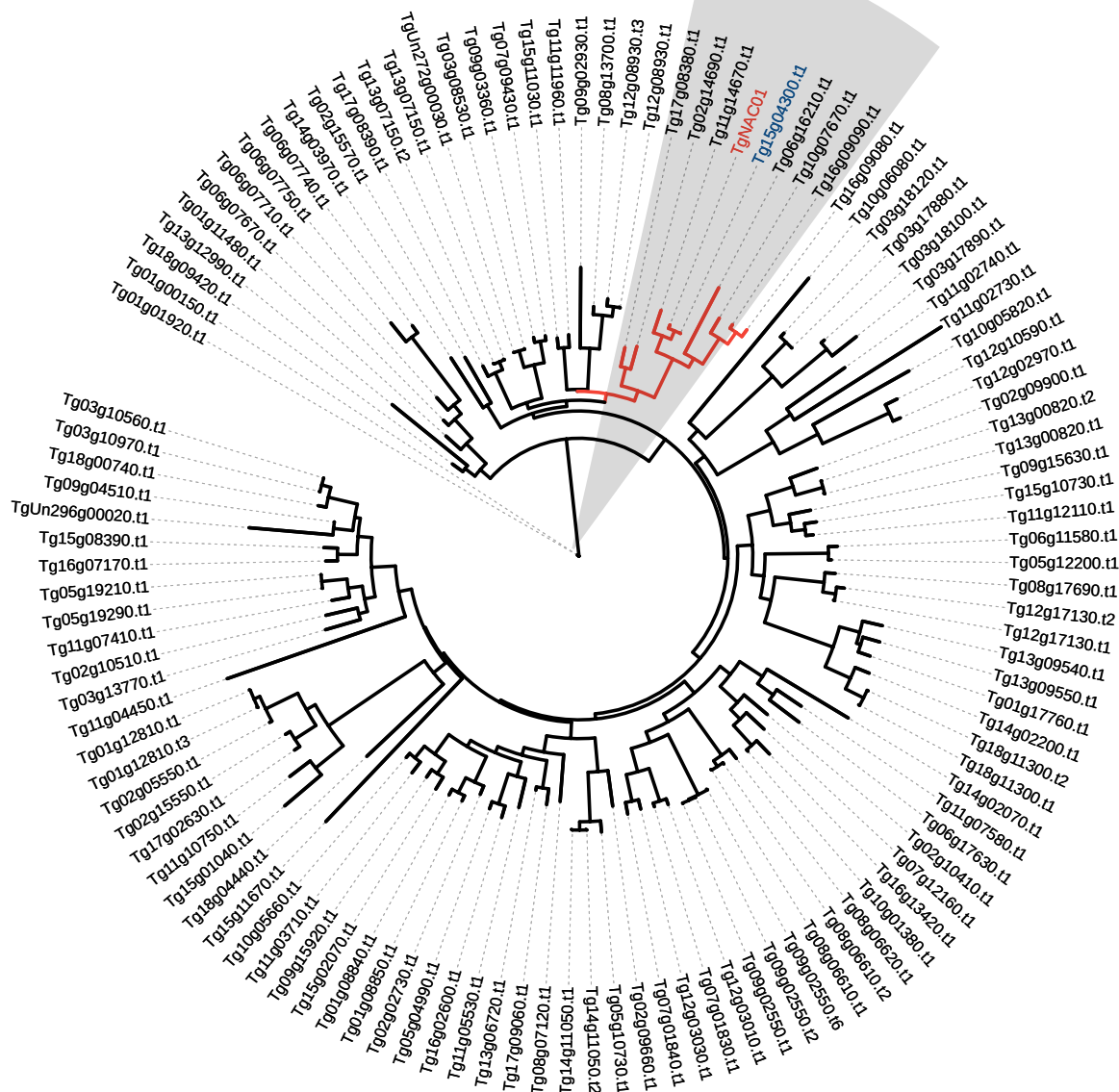

Supplement: Supplemental Information 2 — The phylogenetic tree was constructed using the Likelihood method, pearson correction and 1,000 bootstraps using Mega 7.0. Blue letter is Tg15g04300 and red letter is TgNAC01. [file peerj-10-13039-s002.pdf]

a

35S:TgNAC01

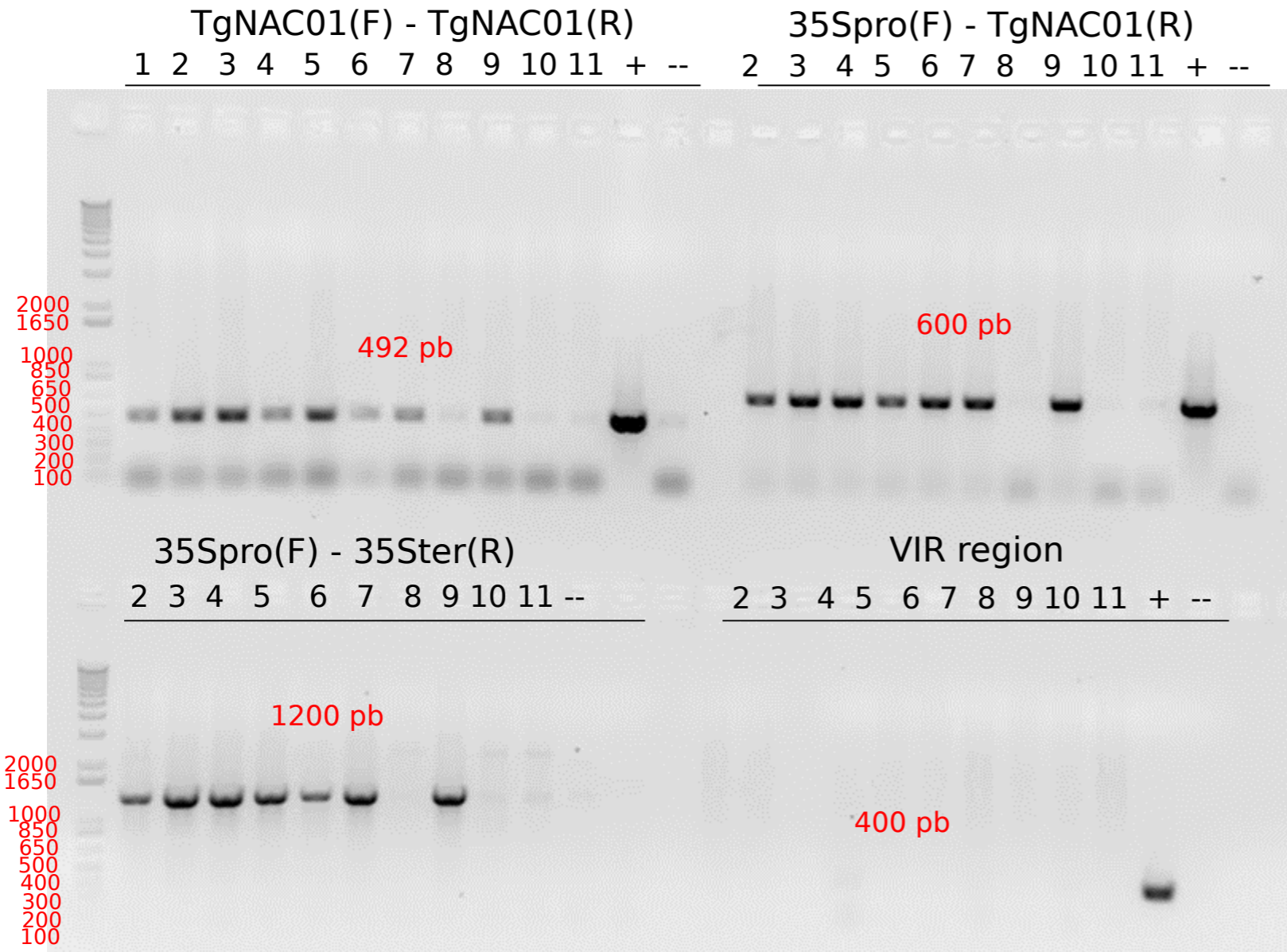

b

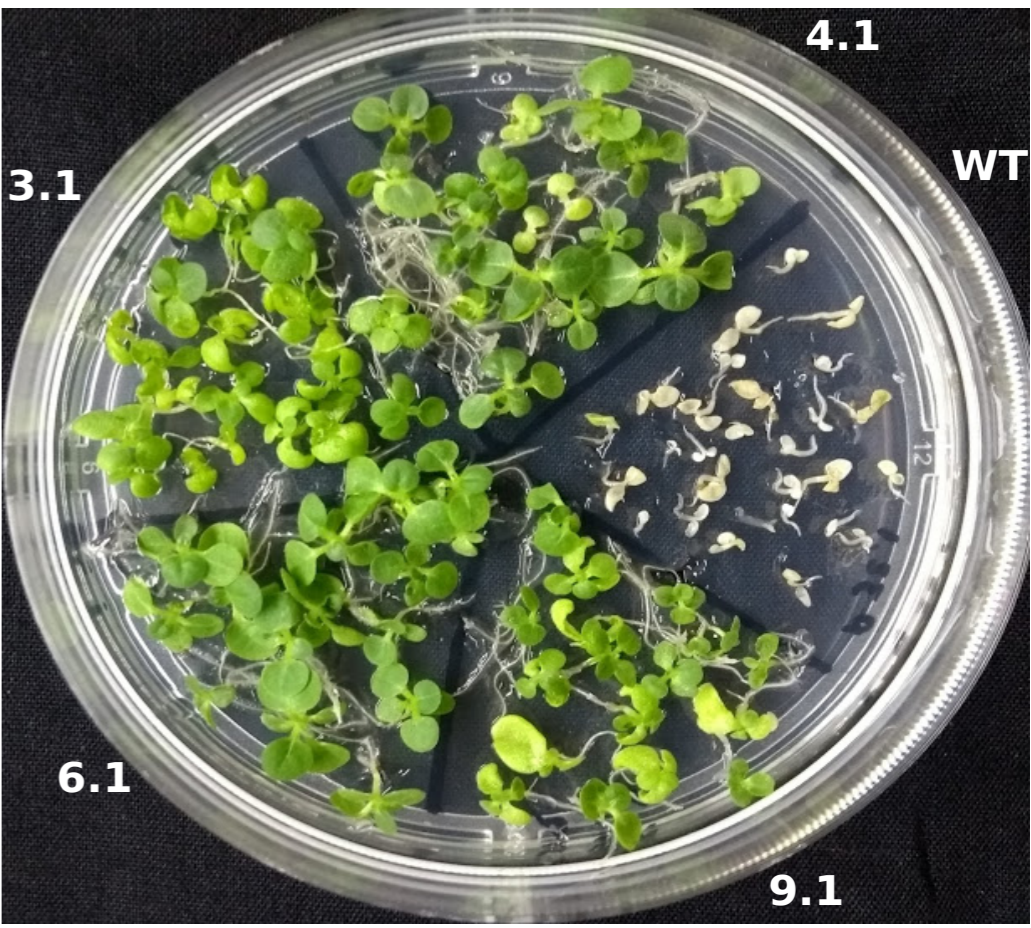

Supplement: Supplemental Information 4 — (A) Agarose gel electrophoresis (1%) of the PCR product corresponding to the 35S:TgNAC01 transgenic lines, using a 1kb Plus DNA Ladder (Invitrogen), with 0.9 µg/lane, 0.9% of agarose stained with ethidium bromide. From lane 1–11: tobacco transgenic lines. Lane 11 corresponds to WT plants. Lane (+) corresponds to the plasmidial DNA from E. coli carrying the 35S:TgNAC01 construction. Lane (-) corresponds to H2O miliQ. (B) Nine-days-old after germination 35S:TgNAC01-3, 35S:TgNAC01-4, 35S:TgNAC01-6, 35S:TgNAC01-9 transgenic lines and WT tobacco seedlings, in a medium selection supplemented with 100 mg L-1 kanamycin [file peerj-10-13039-s004.pdf]

a

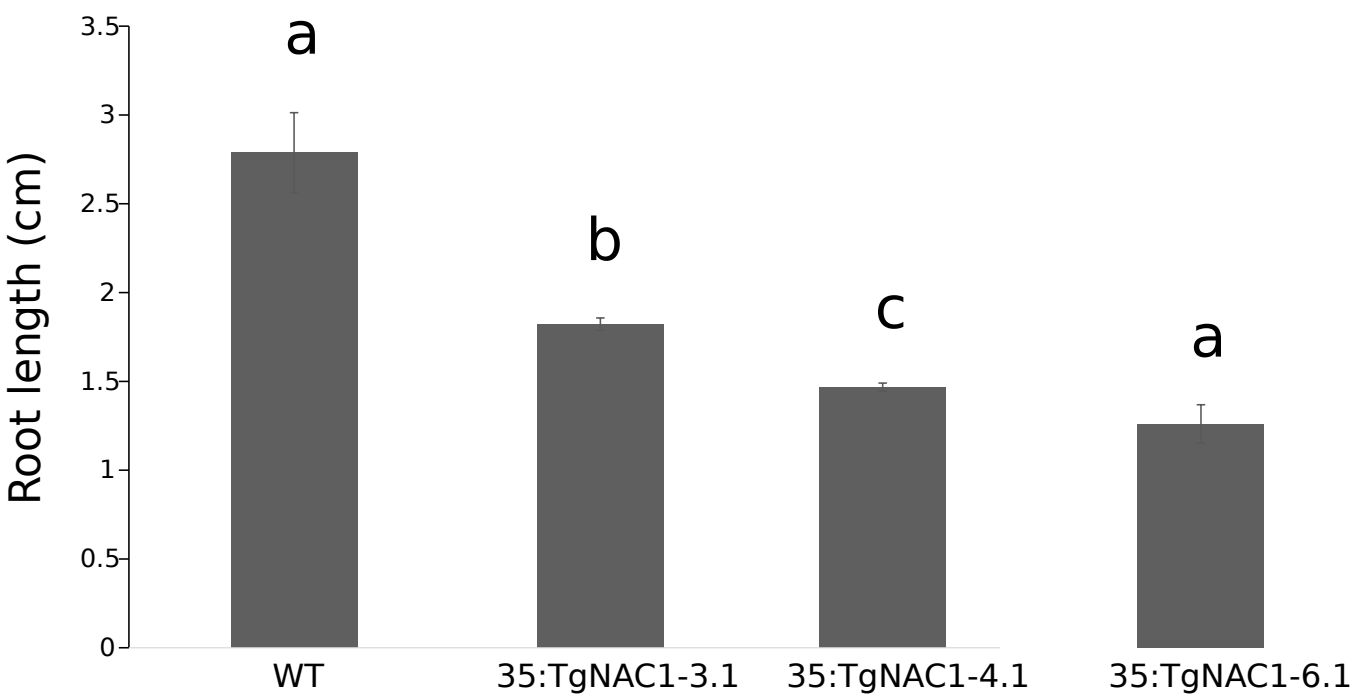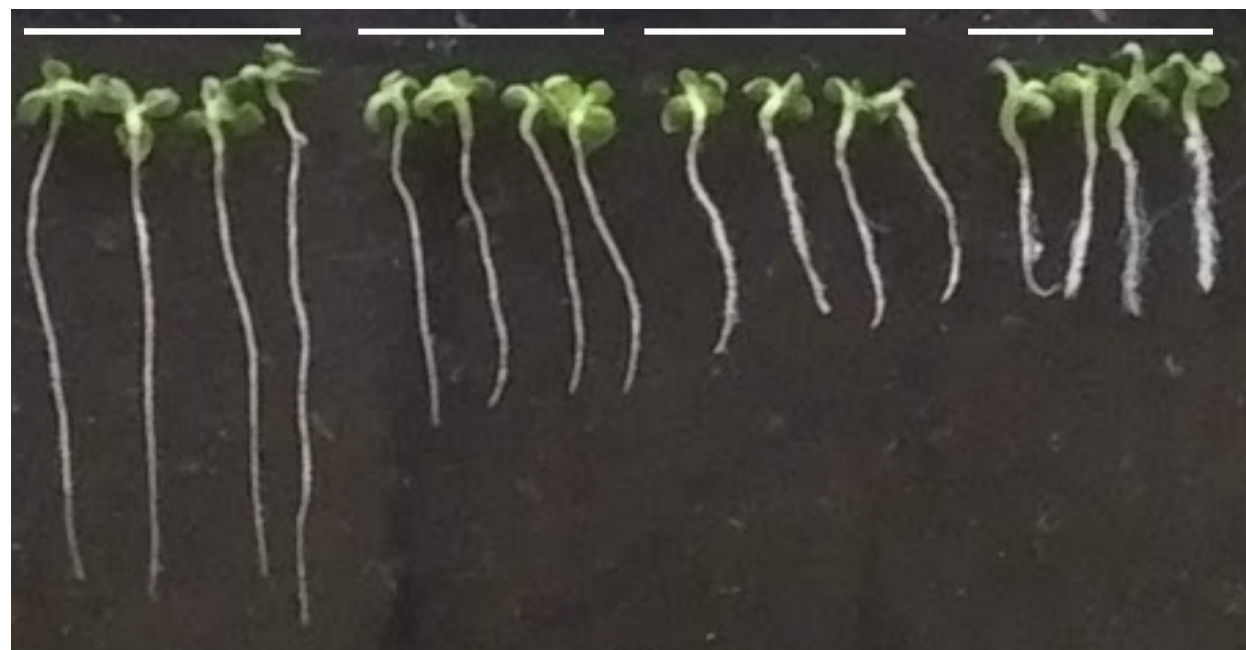

b

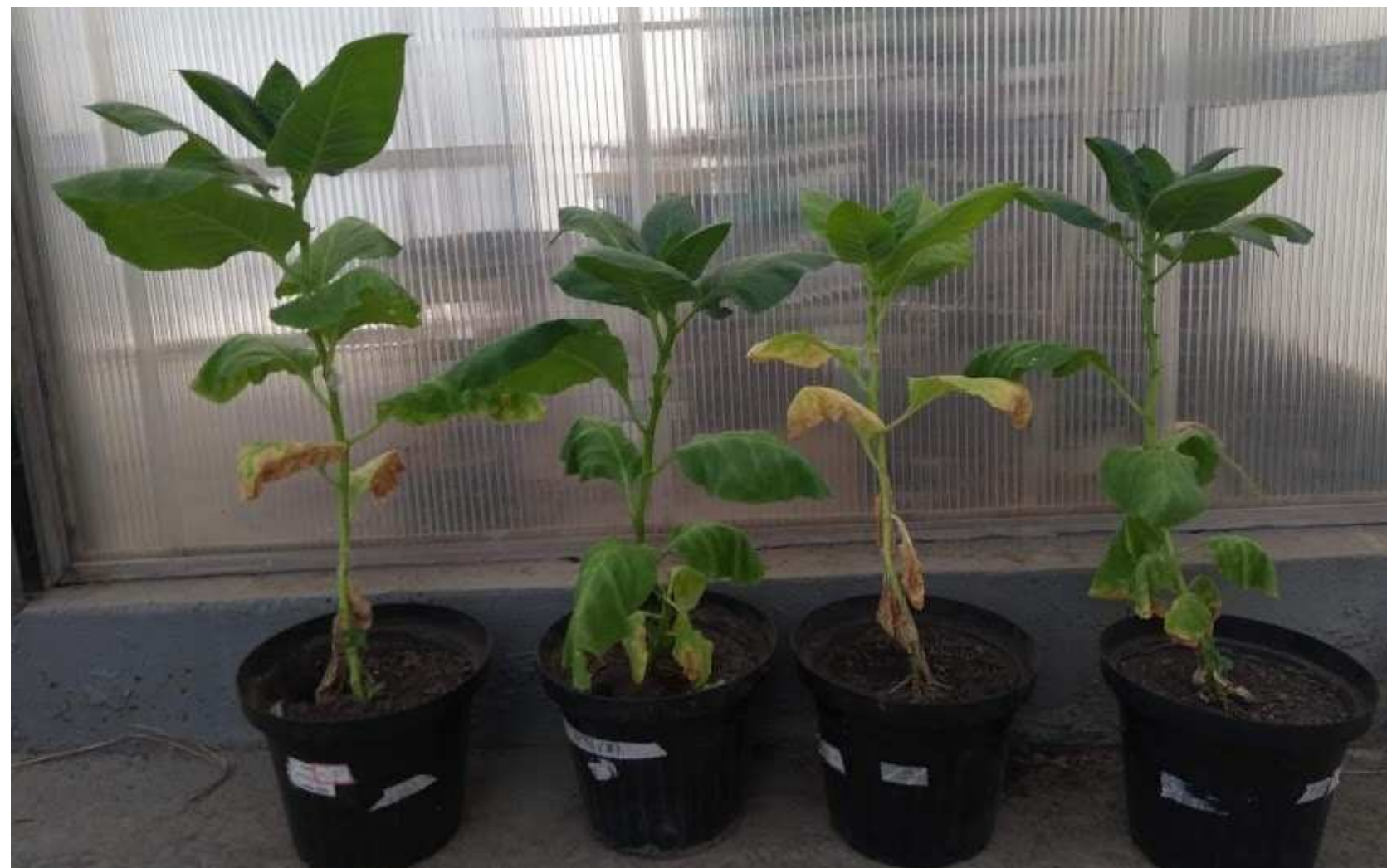35S:TgNAC1-335S:TgNAC1-435S:TgNAC1-6WT

Supplement: Supplemental Information 5 — (A) Root length of 35S:TgNAC01 transgenic lines and WT plants seven days after germination. (B) Phenotype of 35S:TgNAC01 lines and WT at day 60 after germination. Means correspond to three measurements. The standard error of the mean is shown as bars. Letters explain differences between means for root length that 99% of confidence. [file peerj-10-13039-s005.pdf]

# 35S:TgNAC01- EGFP

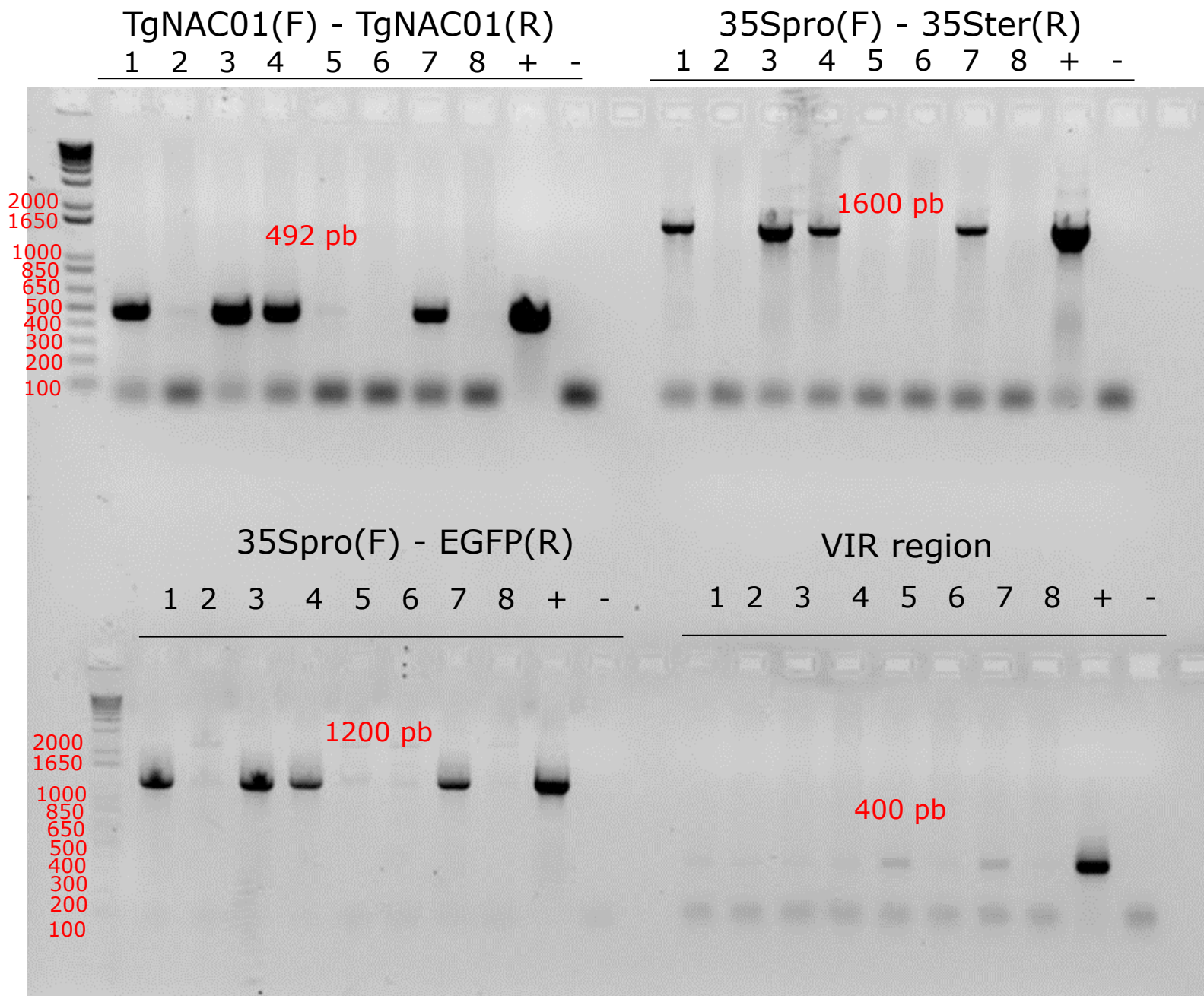

Supplement: Supplemental Information 8 — Agarose gel electrophoresis (1%) of the PCR product corresponding to the 35S:TgNAC01-EGFP transgenic lines, using a 1kb Plus DNA Ladder(Invitrogen), with 0.9 µg/lane, 0.9% of agarose stained with ethidium bromide. From Lane 1–7: tobacco transgenic lines. Lane 8 correspond to WT plants. Lane 9 corresponds to the plasmidial DNA E.coli carrying the 35S:TgNAC01 construction. Lane 10 corresponds to H2O miliQ. [file peerj-10-13039-s008.pdf]

35S:TgNAC1-3

35S:TgNAC1-4

35S:TgNAC1-6

WT

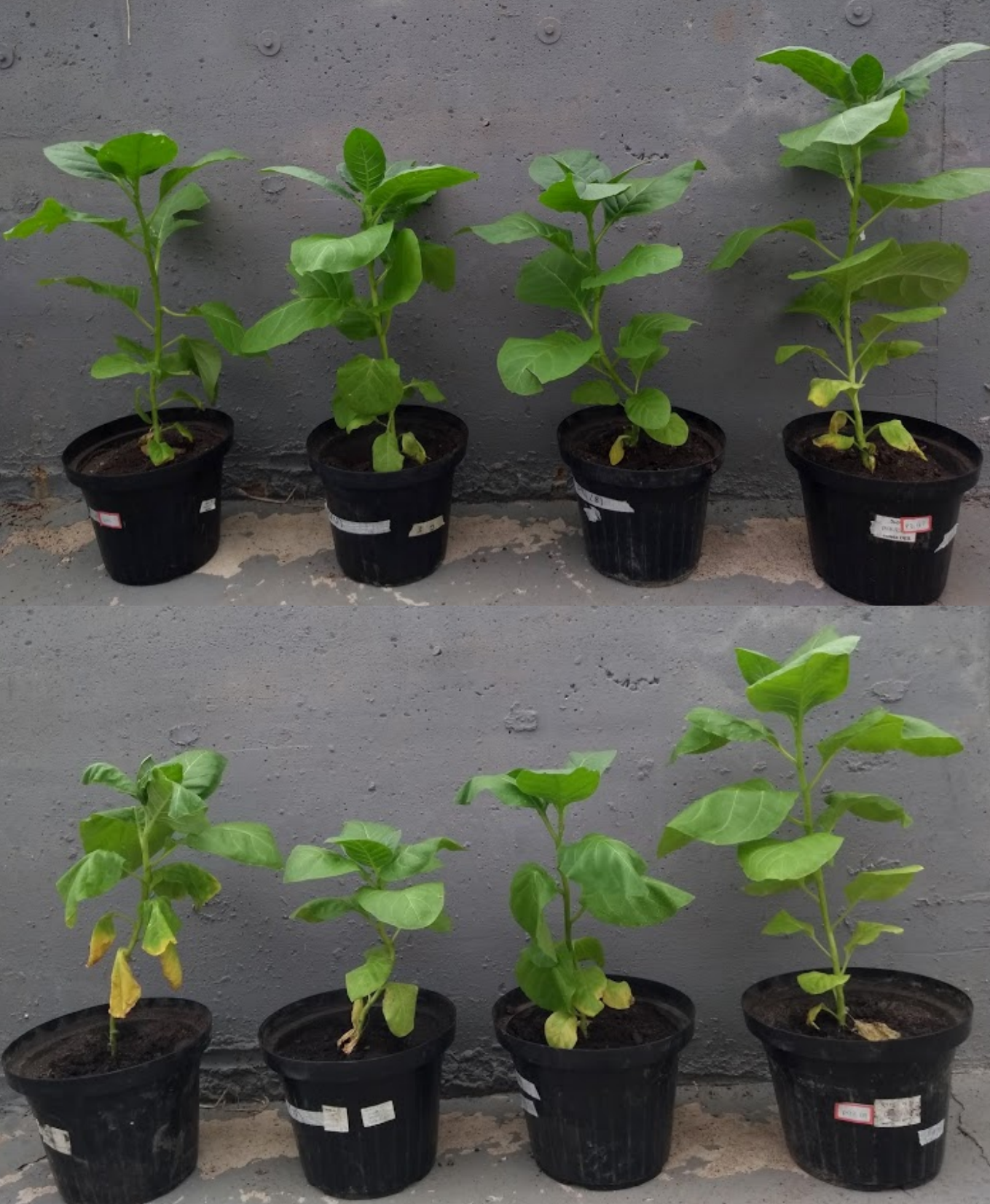

Supplement: Supplemental Information 9 — Phenotype of 35S:TgNAC01 transgenic lines and WT after 7 days under 0 and 300 mM of NaCl concentration [file peerj-10-13039-s009.pdf]
