# Supplementary material for: Overexpression of the Tectona grandis TgNAC01 regulates growth, leaf senescence and confer salt stress tolerance in transgenic tobacco plants
Source: PeerJ. 2022 Mar 3;10:e13039. doi: 10.7717/peerj.13039 (PMC8898551; doi:10.7717/peerj.13039)
Supplement: Supplemental Information 3 — Red letter shows amino acid identity between TgNAC01 and Tg15g04300 [file peerj-10-13039-s003.pdf]

CLUSTAL format alignment by MAFFT (v7.452)

```
TgNAC01      MEKVSLLKNGVLRLPPGFRFHPTDEELVVQYLKRKVLSCPLPASIIPEVDVCKSDPWDLP
Tg15g04300   MEKVSLLKNGVLRLPPGFRFHPTDEELVVQYLKRKVLSCPLPASIIPEVDVCKSDPWDLP
             ************************************************************

TgNAC01      GDSEQERYFFSTREIKYPNGNRSNRATVSGYWKATGLDKQIVSTRSHQIVGMKKTLVFYR
Tg15g04300   GDSEQERYFFSTREIKYPNGNRSNRATVSGYWKATGLDKQIVSTRSHQIVGMKKTLVFYR
             ************************************************************

TgNAC01      GKPPKGCRTDWIMHEYRLITAQNSAITSPQAKNLAQVRFELVA-----------------
Tg15g04300   GKPPKGCRTDWIMHEYRLITAQNSAITSPQAKNLAQENWVLCRIFLKRRNSKTDDEAAAM
             ************************************

TgNAC01      ------------------------------------------------------------
Tg15g04300   QNSGRATGAVPVFYDFMAKERADLNLLPANSSSGSSGITQISSRNESDDHEESSSCNSFP

TgNAC01      ---------
Tg15g04300   TSFRRNLCS
```
