# Supplementary material for: Overexpression of the Tectona grandis TgNAC01 regulates growth, leaf senescence and confer salt stress tolerance in transgenic tobacco plants
Source: PeerJ. 2022 Mar 3;10:e13039. doi: 10.7717/peerj.13039 (PMC8898551; doi:10.7717/peerj.13039)
Supplement: Supplemental Information 6 — Subcellular localization prediction of TgNAC01 protein using Loctree 3 (https://rostlab.org/services/loctree3). [file peerj-10-13039-s006.pdf]

LocTree 3

Protein Subcellular Localization Prediction System

Protein ID	Score	Expected Accuracy	Localization Class	Gene Ontology Terms	Annotation Type
TgNAC01	56	92%	nucleus	nucleus GO:0005634(IDA);	PSI-BLAST

Predicted Localization: nucleus

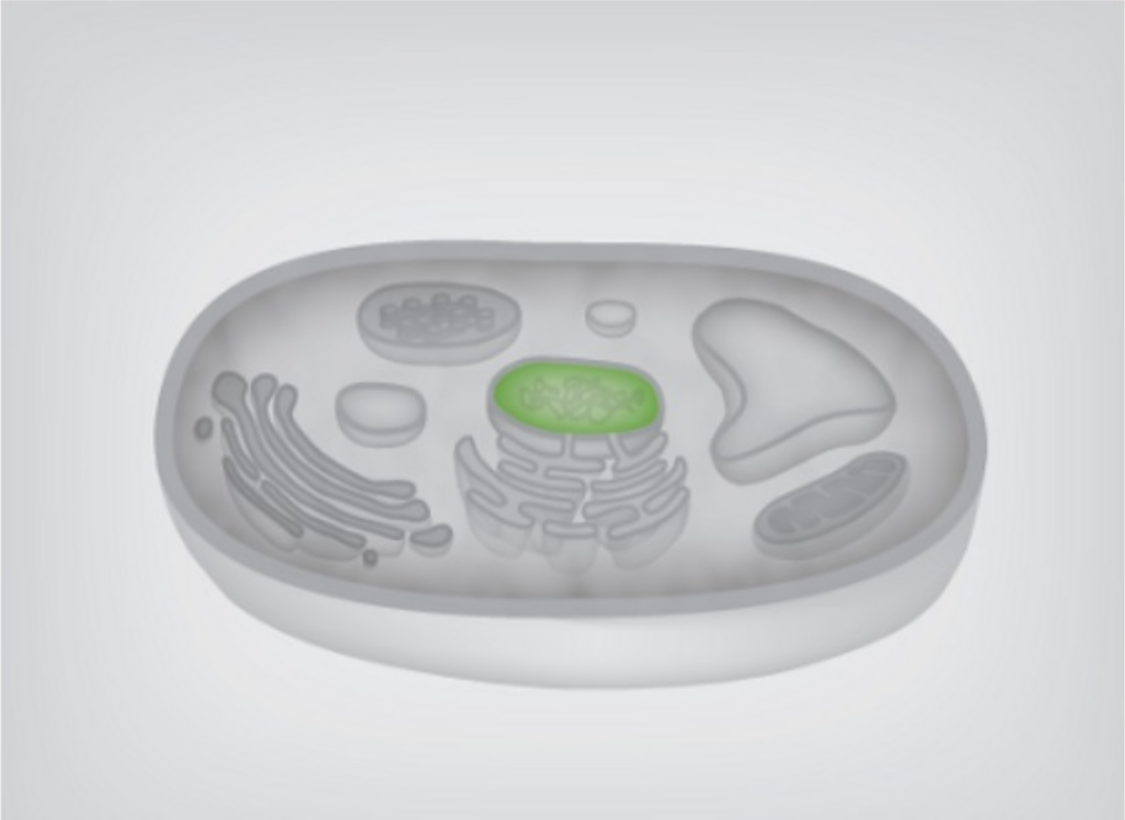

>NAC29_ARATH

```
 Identities = 86/138 (62%), Positives = 101/138 (73%), Gaps = 6/138 (4%)

Query: 11  VLRLPPGFRFHPTDEELVVQYLKRKVLSCPLPASIIPEVDVCKSDPWDLGD----SEQER 66
               LPPGFRFHPTDEEL+V YL+ + +S P P SIIPEVD+ K DPW L +     E E
Sbjct: 6   QSTLPPGFRFHPTDEELIVYYLRNQTMSKPCPVSIIPEVDIYKFDPWQLPEKTEFGENEW 65

Query: 67  YFFSTREIKYPNGNRSNRATVSGYWKATGLDKQIVSTRSHQIVGMKKTLVFYRGKPPKGC 126
            YFFS RE KYPNG R NRA VSGYWKATG DK I S  S+  VG+KK LVFY+G+PPKG
Sbjct: 66  YFFSPRERKYPNGVRPNRAAVSGYWKATGTDKAIHSGSSN--VGVKKALVFYKGRPPKGI 123

Query: 127 RTDWIMHEYRLITAQNSA 144
            +TDWIMHEYRL  ++ ++
Sbjct: 124 KTDWIMHEYRLHDSRKAS 141
```
