# Supplementary material for: Overexpression of the Tectona grandis TgNAC01 regulates growth, leaf senescence and confer salt stress tolerance in transgenic tobacco plants
Source: PeerJ. 2022 Mar 3;10:e13039. doi: 10.7717/peerj.13039 (PMC8898551; doi:10.7717/peerj.13039)
Supplement: Supplemental Information 7 — Transmenbrane predict of TgNAC01 protein using HMMTOP (http://www.enzim.hu/hmmtop/index.php). [file peerj-10-13039-s007.pdf]

Protein: TgNAC01
Length: 163
N-terminus: OUT
Number of transmembrane helices: 0
Transmembrane helices: 0

Total entropy of the model: 17.0131
Entropy of the best path: 17.0131

The best path:

```
seq  MEKVSLLKNG VLRLPPGFRF HPTDEELVVQ YLKRKVLSCP LPASIIPEVD    50
pred OOOOOOOOOO OOOOOOOOOO OOOOOOOOOO OOOOOOOOOO OOOOOOOOOO

seq  VCKSDPWDLP GDSEQERYFF STREIKYPNG NRSNRATVSG YWKATGLDKQ   100
pred OOOOOOOOOO OOOOOOOOOO OOOOOOOOOO OOOOOOOOOO OOOOOOOOOO

seq  IVSTRSHQIV GMKKTLVFYR GKPPKGCRTD WIMHEYRLIT AQNSAITSPQ   150
pred OOOOOOOOOO OOOOOOOOOO OOOOOOOOOO OOOOOOOOOO OOOOOOOOOO

seq  AKNLAQVRFE LVA  163
pred OOOOOOOOOO OOO
```
